# Imaging-Based Prediction of Molecular Therapy Targets in NSCLC by Radiogenomics and AI Approaches: A Systematic Review

**DOI:** 10.3390/diagnostics10060359

**Published:** 2020-05-30

**Authors:** Gaia Ninatti, Margarita Kirienko, Emanuele Neri, Martina Sollini, Arturo Chiti

**Affiliations:** 1 Humanitas University, Pieve Emanuele, 20090 Milan, Italy; gaia.ninatti@st.hunimed.eu (G.N.); arturo.chiti@hunimed.eu (A.C.); 2 Fondazione IRCCS Istituto Nazionale dei Tumori, 20133 Milan, Italy; margarita.kirienko@icloud.com; 3 Department of Translational Research, Diagnostic Radiology 3, University of Pisa, 56126 Pisa, Italy; emanuele.neri@med.unipi.it; 4 Humanitas Clinical and Research Center-IRCCS, Rozzano, 20089 Milan, Italy

**Keywords:** radiogenomics, CT, PET/CT, lung cancer, EGFR, ALK, PD-L1, artificial intelligence, radiomics, targeted therapy

## Abstract

The objective of this systematic review was to analyze the current state of the art of imaging-derived biomarkers predictive of genetic alterations and immunotherapy targets in lung cancer. We included original research studies reporting the development and validation of imaging feature-based models. The overall quality, the standard of reporting and the advancements towards clinical practice were assessed. Eighteen out of the 24 selected articles were classified as “high-quality” studies according to the Quality Assessment of Diagnostic Accuracy Studies 2 (QUADAS-2). The 18 “high-quality papers” adhered to Transparent Reporting of a multivariable prediction model for Individual Prognosis or Diagnosis (TRIPOD) with a mean of 62.9%. The majority of “high-quality” studies (16/18) were classified as phase II. The most commonly used imaging predictors were radiomic features, followed by visual qualitative computed tomography (CT) features, convolutional neural network-based approaches and positron emission tomography (PET) parameters, all used alone or combined with clinicopathologic features. The majority (14/18) were focused on the prediction of epidermal growth factor receptor (EGFR) mutation. Thirty-five imaging-based models were built to predict the EGFR status. The model’s performances ranged from weak (*n* = 5) to acceptable (*n* = 11), to excellent (*n* = 18) and outstanding (*n* = 1) in the validation set. Positive outcomes were also reported for the prediction of ALK rearrangement, ALK/ROS1/RET fusions and programmed cell death ligand 1 (PD-L1) expression. Despite the promising results in terms of predictive performance, image-based models, suffering from methodological bias, require further validation before replacing traditional molecular pathology testing.

## 1. Introduction

Primary lung cancer is the most common malignancy worldwide, accounting for 11.6% of all cancers and 18.4% of all cancer-related deaths. In 2018, more than 2 million people were diagnosed with lung cancer globally and more than 1.75 million deaths occurred [1].

Non-small cell lung cancer (NSCLC) accounts for 80–90% of all primary lung cancers, and the majority of patients have an advanced stage unresectable disease at diagnosis, which carries a dismal prognosis.

In recent years, unprecedented advancements in the management of lung cancer have been made. The increasing understanding of the molecular and genetic alterations at the basis of NSCLC and of the mechanisms of immune evasion by cancer cells have paved the way for novel targeted drugs and immunotherapeutic agents [2]. Because of this, histological diagnosis nowadays needs to be complemented by accurate molecular profiling, which is aimed at detecting biomarkers for personalized treatment selection (Table 1).

Currently, testing for molecular alterations that serve as robust targeted therapy-predictive biomarkers is recommended to guide treatment selection in patients with advanced and metastatic adenocarcinoma. In particular, testing for epidermal growth factor receptor (EGFR), tyrosine kinase receptor (ALK) as well as ROS1 and BRAF oncogene mutation should be routinely performed, while testing for other oncogenes such as RET, HER2, KRAS and MET is indicated only in selected cases [3,4,5].

Molecular-targeted therapy with tyrosine kinase inhibitors (TKIs) specifically directed to these alterations has been shown to improve patient outcomes both in terms of survival and drug-induced toxicities compared to standard chemotherapeutic agents [6,7,8,9,10,11,12,13,14,15,16,17,18,19,20,21].

Immune-checkpoint inhibitors (ICIs) for the treatment of advanced NSCLC have been recently approved. The percentage of tumor cells expressing programmed cell death ligand 1 (PD-L1) at immunohistochemistry is the routinely used biomarker to select candidates for this additional therapeutic option [5]. The PD-1/PD-L1 inhibitors have indeed been successful in improving survival, particularly in patients without targetable molecular alterations [22,23,24,25,26,27]. This benefit in terms of overall survival was especially noticed in patients with ≥50% of tumor cells expressing PD-L1 at immunohistochemical analysis [23].

To sum up, advancements in the field of molecular pathology have allowed the stratification of NSCLC patients according to oncogenic driver mutations and PD-L1 expression, with a huge impact on treatment tailoring. However, testing to identify therapy-predictive biomarkers currently relies on the analysis of tumor samples collected from conventional biopsies or cytological specimens, which carry some inherent limitations. These indeed are invasive procedures that are not always feasible, often result in the collection of inadequate samples and cannot capture intra- and inter-tumor heterogeneity, being representative of only a minor portion of the malignancy. Moreover, in the case of disease recurrence after first-line treatment, re-biopsy is not mandatory and targeted therapies may be offered based on the detection of molecular alterations tested on the surgical specimens, assuming that no molecular variations occur between primary tumor and recurrence [34].

In this scenario, the need of complementing or replacing traditional testing based on tissue biopsy and cytology samples with other methods able to assess actionable biomarkers in NSCLC is emerging.

In this context, imaging, performed for baseline staging and response evaluation in lung cancer, is gaining a renewed interest as a potential biomarker for non-invasive tumor characterization, since the introduction of radiomics and artificial intelligence (AI). Radiomics is a process of extraction and the analysis of quantitative features (or quantitative imaging biomarkers) from diagnostic images. The process of radiomics extracts intrinsic digital features of tissues that are not perceivable by human interpretation and in oncologic applications, tumor heterogeneity is of major interest. The heterogeneity of tumoral tissue may correlate with aggressiveness and response to treatment. Most clinical potential applications of radiomics are in the prediction of the response to treatment. However, of particular interest is the radiogenomic approach, which aims to assess the correlation between quantitative imaging features and genomic profiles. The underlying hypothesis of radiogenomics is that the quantitative imaging features can capture gene-expression patterns, representing, therefore, the phenotype of the genomic signature. Radiogenomics is particularly attractive since it represents a non-invasive, repeatable, fast and cost-effective method of extracting molecular information from images. Even more recently, AI-based approaches have been applied to medical imaging. These approaches may be used in combination with radiomics or stand-alone. The main advantage of AI-based approaches is in the identification of relevant features in a data-driven fashion. On the other hand, in the majority of cases, it is impracticable to go backwards from the output to the input to interpret final results, being the “black-box” one of the main issues of these approaches [35,36,37,38,39,40,41,42,43,44,45].

The objective of this study was to analyze the current state of the art of imaging-derived biomarkers predictive of genetic alterations and immunotherapy targets in NSCLC by using a systematic literature review.

## 2. Materials and Methods

### 2.1. Eligibility Criteria, Search Strategy and Study Selection

A comprehensive literature search for potentially relevant papers published up until 12 February 2020, was performed using the PubMed/MEDLINE database. No limitations on the publication date were applied. The search strategy combined terms referring to “radiogenomics”, “lung cancer”, “molecular alterations/targeted therapy/PD-1” as well as “PD-L1/immunotherapy” and “imaging” in order to identify the relevant papers for the topic. Details on the search terms are reported in the Supplementary Materials.

Subsequently, additional research studies of possible interest were identified from the reference lists of the retrieved articles and reviewed for eligibility. Additional potentially relevant records were searched on ClinicalTrials.gov (https://clinicaltrials.gov) [46].

Original research studies reporting the creation of imaging features-based models for the prediction of PD-L1 expression or the presence of targetable mutations in NSCLC were included.

After the removal of the duplicates, the titles and abstracts of retrieved records were screened the following exclusion criteria were applied: (1) full text not available in English; (2) review articles, editorials, commentaries, case reports; (3) studies performed on non-humans; (4) studies involving <20 subjects; and (5) the articles not within the field of interest.

The full text of the remaining articles was then screened with the following exclusion criterion: (6) descriptive or exploratory studies with neither internal (e.g., bootstrapping and cross-validation) nor external (e.g., split-sample, temporal or another institution cohort) validation of the predictive model.

### 2.2. Analysis of Quality and Reporting Completeness

The quality of each included study was assessed using the Quality Assessment of Diagnostic Accuracy Studies 2 (QUADAS-2) criteria [47], which comprises four domains. The QUADAS-2 domains “patient selection”, “index test” and “reference standard”, assess both the risk of bias and applicability, while the “flow and timing” domain assesses the risk of bias only.

According to the scope of the present systematic review, studies with adherence to QUADAS-2 ≤ 4/7 were classified as “high/unclear risk of bias” or as having “high/unclear concerns regarding applicability” and consequently, were not considered in the quantitative analysis.

As for the completeness of the reporting, the Transparent Reporting of a multivariable prediction model for Individual Prognosis Or Diagnosis (TRIPOD) checklist [48] was applied to each included article. A TRIPOD checklist adapted to radiomic studies proposed by Park et al. [49] was used to assess the radiomic and AI studies.

The analyses of the quality and completeness of the reporting were performed independently by two reviewers (G.N. and M.S.).

### 2.3. Data Extraction and Analysis

A summary of the study characteristics (i.e., qualitative analysis) was done, including all the selected papers. A quantitative analysis was instead performed only taking into consideration “high-quality papers”, defined as those studies neither at significant risk of bias nor with applicability problems according to the criteria mentioned above.

For the quantitative synthesis, the study characteristics, the main results with metrics (area under the curve, AUC, or other measures of diagnostic accuracy, including sensitivity, specificity, and accuracy) and the TRIPOD overall adherence rate were collected within a database. The following study characteristics were recorded: year of publication, type of study (prospective or retrospective), number of included subjects and their ethnicity, histological subtype and stage for each patient, imaging modality (CT or PET/CT), the molecule of interest (EGFR, ALK, ROS1, BRAF, RET, KRAS, HER2, MET, or PD-L1), the type of imaging features used for predictions, either imaging features (visual qualitative CT features, radiomic features, PET parameters or convolutional neural network-based approaches) or clinicopathologic features, and the type of validation (internal or external).

If a study had two or more molecules of interest or investigated the predictive potential of two or more types of imaging features, it was considered as two or more separate studies.

If a study used two or more different types of prediction algorithms (e.g., logistic regression, support vector machines, random forest), the main results were reported only for the model with the best performance.

Descriptive statistical metrics were used to summarize the data.

The studies were gathered according to the molecule of interest and grouped based on the TRIPOD adherence rate. Accordingly, we established different levels of adherence to TRIPOD (i.e., very low, low, moderate, high, and very high) setting a 10% incremental value from 50% to 100%, and each study was ranked from the high-to-low level of the quality, based on the assumption that the higher the TRIPOD adherence rate, the stronger the investigation. Subsequently, the performance of each model was assessed using the metrics mentioned above. The area under the curve (AUC)—whenever available—was preferred to other metrics (e.g., sensitivity) to summarize the diagnostic accuracy of the proposed model. The AUCs were rated as null (0.50–0.60), poor (0.60–0.70), acceptable (0.70–0.80), excellent (0.80–0.90) and outstanding (>0.90) [50,51]. A trial phase from I to IV was assigned to each study in order to assess how far it is from clinical practice [52,53]. Excel^®^ 2017 (Microsoft^®^, Redmond, WA, USA) was used for the analysis.

## 3. Results

### 3.1. Study Selection

The search of the PubMed/MEDLINE database returned a total of 563 studies (methods detailed in Section 2). By screening the cited articles of the retrieved papers, 10 additional studies that met inclusion criteria were identified. None of the 10 articles selected from reference lists passed the selection phase, being all without validation. No relevant record pertinent to the review’s topic was found in ClinicalTrials.gov (https://clinicaltrials.gov). After the removal of duplicates, 549 records were left. After the abstract review, 473 studies were excluded. The screening process is summarized in Supplementary Figure S1. Twenty-four articles were finally included and assessed for quality.

### 3.2. Study Characteristics and Risk of Bias within Studies

The 24 selected articles were retrospective studies developing multivariable models for the prediction of molecular genetic alterations (*n* = 22) or PD-L1 expression (*n* = 2). Seventeen studies aimed at predicting EGFR status [54,55,56,57,58,59,60,61,62,63,64,65,66,67,68,69,70], one aimed at predicting ALK status [71], three at predicting both EGFR and KRAS status [72,73,74], one at identifying ALK/ROS1/RET fusion-positive versus fusion-negative adenocarcinomas [75] and two at predicting the PD-L1 expression level [76,77]. Study characteristics are summarized in Table 2. Supplementary Table S1 provides details of the molecular genetic alterations or PD-L1 expression stratified according to the stage (early versus advanced).

After assessment through the Quality Assessment of Diagnostic Accuracy Studies 2 (QUADAS-2) criteria (Supplementary Figure S2), six out of the 24 (25%) [65,66,67,72,73,74] selected studies did not reach the score for “high-quality papers” (QUADAS-2 > 4/7). In particular, five studies [65,66,72,73,74] had a high/unclear risk of bias, most commonly in the “patient selection” and “index test” categories and one study [67] had applicability problems in the “patient selection” category.

**Figure 1:**
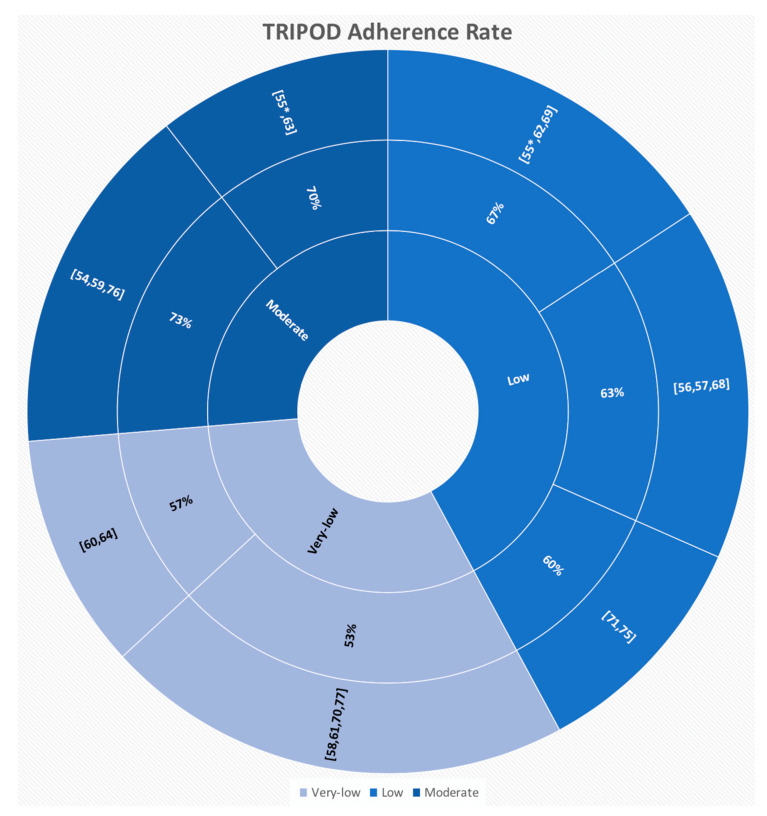
Adherence to the Transparent Reporting of a multivariable prediction model for Individual Prognosis or Diagnosis (TRIPOD) for the “high-quality papers”.

The 18 “high-quality papers” varied in terms of adherence to Transparent Reporting of a multivariable prediction model for Individual Prognosis or Diagnosis (TRIPOD), ranging from 53% to 73% (mean = 62.9 ± 7.2% standard deviation), as detailed in Figure 1.

For all these investigations, the TRIPOD adherence rate did not change in the case of multiple molecules of interest or types of imaging features investigated within the same study. In one case (i.e., [55]) the calculated TRIPOD adherence rate differed based on the investigated types of imaging features, being 70% for the radiomics-based model (i.e., moderate) and 67% for the qualitative features-based model (i.e., low), respectively.

Two studies were classified as phase I [60,68], and the remaining 16 investigations as phase II (IIa = 2 [64,76], and IIb = 14 [54,55,56,57,58,59,61,62,63,69,70,71,75,77]).

### 3.3. Main Results

#### 3.3.1. Prediction of EGFR Status

Out of the 18 “high-quality papers”, 14 focused on the prediction of EGFR mutation [54,55,56,57,58,59,60,61,62,63,64,68,69,70].

**Figure 2:**
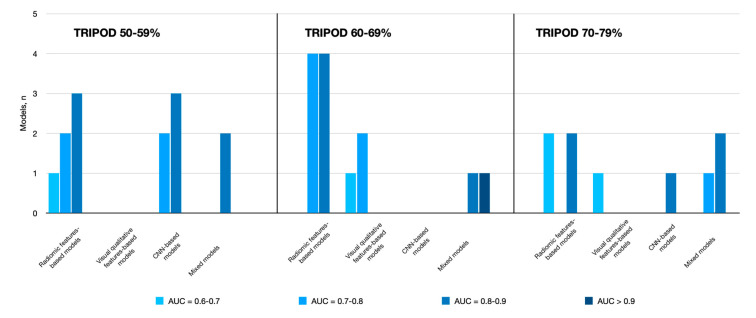
Summary of the performances for the models aiming at predicting EGFR status, divided according to the method.

Thirty-five predictive models were built, considering all 14 studies. The predictive ability of the different types of imaging-based models for EGFR status is summarized in Figure 2. Of these, the majority were radiomics-based models (*n* = 18), with (*n* = 6 [55,56,57,58,62,64]) or without (*n* = 12 [54,55,56,57,58,59,60,62,64,68,69,70]) the addition of clinicopathological features. The area under the curve (AUC) values in the validation cohorts ranged from 0.64 to 0.89 (details are provided in Supplementary Table S2). When added to radiomic features, the clinical parameters brought an improvement in the classification performance in one out of six cases (AUCs of 0.77 and 0.87 for radiomics and radiomics + clinical, respectively [62]). In the remaining five cases, the AUCs of both radiomics and radiomics + clinical models fell in the same rank (acceptable = 2 [56,58], and excellent = 3 [55,57,64]). Of note, the two radiomics-based models that adhered the most to TRIPOD reported unsatisfactory AUCs [54,59]. Conversely, the great majority of radiomics-based investigations adherent to TRIPOD at the very-low level showed good model performance [58,60,64]. Studies using radiomic models, alone or combined with clinical models, to predict EGFR status are summarized in Table 3.

Four predictive models were instead based on visual qualitative computed tomography (CT) features, together (*n* = 2 [55,69]) or not (*n* = 2 [59,68]) with clinicopathologic features (Table 4). The AUC range in the validation cohorts was 0.62–0.77. The visual qualitative CT features most commonly associated with EGFR mutation are reported in Table 5.

An additional six models were convolutional neural network (CNN)-based approaches, again combined (*n* = 2 [58,61]) or not (*n* = 4 [58,59,61,70]) with clinical models. The AUC values in the validation groups ranged from 0.75 to 0.84, and all the models benefited from the addition of clinicopathologic features, particularly the model proposed by Xiong et al. [61] (the AUC improved from acceptable to excellent). Five out of six models had a very low adherence to TRIPOD (Table 6).

Finally, seven models based on different combinations of radiomic features, visual qualitative CT features, convolutional neural network (CNN)-based approaches, positron emission tomography (PET) parameters and clinicopathologic features were reported. Among these, the lowest AUC in the validation cohort was 0.73 [54]. The predictive model with the highest AUC in the validation set (AUC = 0.95) resulted from the combination of radiomic and visual qualitative CT features [68]. Two out of seven combined models—within the same study [58] and both resulting in an excellent performance—were rated adherent to TRIPOD at a deficient level. The details of studies using combined models to predict EGFR status are reported in Table 7.

The number of variables included in the models significantly varied (range 2–32, mean 9 ± 8 standard deviation) regardless of the type (i.e., radiomic or visual qualitative). Moreover, the selected radiomics features were not listed [57,59,68] or clearly reported [58,70], resulting in an incomplete reporting of the model in approximately 40% of cases (7/18 radiomics-based models and 3/7 combined models, respectively). Conversely, all the studies evaluating the performance of visual qualitative image analysis (alone or combined with other types of imaging features), specified the features included in the models. Nonetheless, when radiomic or visual qualitative features were detailed, the models resulted as inconsistent among the investigations. No more than two of the selected features were the same in more than two models. The number and type of clinicopathological features were less variable (range 1–5, mean 2 ± 1 standard deviation) than the imaging features among analyzed investigations. Particularly, sex and smoking commonly entered in models, being tested for their association with EGFR status in 93% and 68% of cases, respectively. Clinical features most commonly associated with EGFR mutation are reported in Table 8.

Notably, 38% of the investigations compared at least two types of imaging features in predicting the EGFR status [54,55,58,59,68,69,70]. As expected, the CNN-based approaches outperformed radiomics-based models [58,70]. The only study that tested radiomic versus visual qualitative versus CNN-based approaches confirmed that deep learning outperformed both radiomic and CT-features (AUCs of 0.81 versus 0.64 and 0.64, respectively), and it showed that radiomic analysis did not offer any advantage over visual qualitative analysis [59]. These data differed from those reported by Lu et al. [55], Jiang et al. [68] and Tu et al. [69]. They indeed showed that radiomic models performed better than visual qualitative CT feature-based models [55,68,69] and that the models’ performances were further powered when both the approaches were combined [68,69].

#### 3.3.2. Prediction of EGFR Mutation Subtypes

One study by Zhao et al. [54] aimed at predicting the subtype of EGFR mutation, in particular the two most common ones (exon 19 deletion and exon 21 L858R mutation), using both a radiomics-based model and a combined radiomic and clinical model. The respective AUC values in the validation cohort were 0.71 and 0.76. The details of these models are reported in Table 9.

#### 3.3.3. Prediction of ALK Status and ALK/ROS1/RET Fusions

Yamamoto et al. [71] aimed instead at predicting the ALK status using visual qualitative CT features combined with clinical parameters. Their predictive model had a good performance in both the training and the validation set (Table 10 and Supplementary Table S2).

Another study by Yoon et al. [75] investigated the potential of the combined radiomic features, the PET parameters, the visual qualitative CT features, and the clinical data to differentiate the ALK/ROS1/RET fusion-positive and fusion-negative adenocarcinomas, building a model that resulted in 73% sensitivity and 70% specificity in the 10-fold cross validation (Table 11).

#### 3.3.4. Prediction of PD-L1 Expression Levels

The two remaining investigations out of the 18 included studies, by contrast, focused on the PD-L1 expression levels prediction, as detailed in Table 12.

Jiang et al. [77] aimed to predict both PD-L1 expression level ≥1% and ≥50% using radiomics features exclusively and built a model with the highest AUC = 0.97 and 0.91, respectively, for the two tasks (Supplementary Table S2). Interestingly, the same combination of radiomic features was revealed to be able to predict both the PD-L1 expression level ≥1% and ≥50%.

On the other hand, Yoon et al. [76] aimed to predict only the PD-L1 expression level ≥50% using a radiomics- and clinical features-based model with an AUC = 0.67, applying the bootstrapping approach (Supplementary Table S2).

## 4. Discussion

The present systematic review evaluated the results, the overall quality, the standard of reporting, and advancement towards the clinical practice of the investigations aimed at evaluating imaging-derived biomarkers to predict genetic alterations and immunotherapy targets in NSCLC. Other systematic reviews have been published on radiogenomics in lung tumors [78,79,80]. However, to the best of our knowledge, this is the first that includes radiogenomics, conventional analysis (visual qualitative CT analysis and PET parameters), and AI-based approaches, assessing the predictive ability of imaging-derived biomarkers in terms of their reliability, robustness and clinical implementability.

CT and PET imaging-derived radiomic features, CNN-based approaches, PET parameters, and visual qualitative CT features were tested for the prediction of actionable mutations. Most of the published studies were focused on EGFR alterations, which are the most commonly encountered actionable mutations in clinical practice, being present in 40–50% and 10–20% of NSCLC patients of Asian and non-Asian ethnicity, respectively [33,81,82]. The imaging-based predictive models were able to predict EGFR status, with performances ranging from poor (AUC = 0.6 to 0.7, *n* = 5) to acceptable (AUC = 0.7 to 0.8, *n* = 11), excellent (AUC = 0.8 to 0.9, *n* = 18), and outstanding (AUC > 0.90, *n* = 1) in the validation set. However, as mentioned previously, the AUC of a model is not itself informative, since many other significant items, each contributing for a predetermined rate, account for the reliability of a study. Positive outcomes were also reported for the prediction of other molecular alterations, including ALK rearrangement and ALK/ROS1/RET fusions. However, very few studies have been published with this aim, and more advanced image analyses are thus needed to confirm these preliminary results. The majority of models (67%) were validated using an independent set of patients through the split-sample approach. The geographic validation was done in only one case (5%). However, the latter should be preferred. Benefitting from technical variability aspects, it measures better the model’s performance and provides a proof of generalizability [52,83]. Cross-validation was the most frequent method used in the case of internal validation (4/5 models).

Given the models’ performance, it is evident that advanced image analysis techniques as radiomics and AI are the most promising methods in the field of tumor phenotyping.

Targeted therapies are offered to patients with advanced/metastatic NSCLC. All the included papers but the one by Yamamoto et al. [71] reported results of molecular alterations regardless of the stage of the disease (i.e., including patients with early disease stage and/or without stratification according to stage). This is to some extent consistent with the common practice. Indeed, targeted therapy indication can be based on the detection of molecular alterations tested on the surgical specimens obtained at primary surgery or diagnostic biopsy. This practice relies on the assumption that no or minor molecular variations occur between primary tumor and recurrence, and therefore between early and advanced stages. Nonetheless, future investigations should take into account the molecular alteration landscape at different stages of the disease.

The predictive potential of PET parameters and visual qualitative features was investigated. Zhang et al. [63] found that a lower peak standardized uptake value (SUV_peak_) was associated with EGFR mutations, while spiculation, the absence of emphysema, pleural indentation and the subsolid nodule were the semantic CT features most commonly associated with EGFR mutations. However, there are no standardized definitions for visual qualitative features, and this may affect the reproducibility of results. Radiomics provides objective, repeatable and quantitative assessments. On the other hand, the possibility of analyzing images with “intelligent” methods (e.g., unsupervised), and the development of strategies to address the “black-box” and accountability issues [35] make CNN-based approaches even more attractive in the field of medical imaging.

Reported results suggest that combining different methods for image biomarker extraction may help to improve the predictive performances of the models and be a winning strategy towards their implementation into clinical practice. Particularly useful were the combinations of (1) CT and PET radiomic features and PET parameters; (2) CT radiomic features and visual qualitative CT features; and (3) CT radiomic features and CNN-based approaches. The potential of combined models, therefore, needs to be investigated further with future studies. Moreover, the importance of adding clinical features to improve the performance of imaging-based predictive models must be underlined. For example, most of the included studies reported a statistically significant association of the female sex and non-smoking status with EGFR mutation. These findings were consistent with large-scale molecular epidemiological investigations that were done in patients affected by NSCLC [82,84]. Nonetheless, we did not find common, reliable radiomic features among the studies. This finding may be related to the different tools applied for feature calculation and different approaches to data analysis.

Coming to biomarkers of immunotherapy response, two studies among the selected ones successfully predicted PD-L1 expression level using radiomic features, alone or together with clinicopathologic characteristics [76,77]. However, the reliability of PD-L1 expression as a biomarker is a matter of current debate [85] and this will have to be taken into account for future imaging studies.

PD-L1 expression is indeed dynamic and variable, and this depends on many different factors, both tumor-dependent (heterogeneity of PD-L1 expression within and between tumor lesions, PD-L1 expression by various cell types in the tumor microenvironment) and immunohistochemistry assays-dependent (different antibodies used to detect PD-L1, variable cut-offs to define a PD-L1 test result as positive) [86,87]. Even if PD-L1 expression is associated with an increased likelihood of response to immunotherapy, there are cases of non-responsive PD-L1 positive tumors and responsive PD-L1 negative tumors [87]. Accordingly, the potential of other biomarkers is being explored and imaging studies will have to adapt to possible future changes in biomarker testing for immunotherapy in NSCLC.

According to our assessment, the quality of the studies resulted unsatisfactory and the reporting was incomplete. Therefore, the proposed models are to be considered immature for clinical implementation.

QUADAS is a tool developed to assess the quality of diagnostic accuracy investigations [88]. We found that 25% of the selected studies did not reach the score for “high-quality papers” (QUADAS-2 > 4/7), being affected by high/unclear risk of bias, most commonly in the “patient selection” and “index test” categories.

We further assessed the quality of the studies evaluating the TRIPOD adherence rate for each model built to predict genetic alterations and immunotherapy targets. The exhaustive and careful reporting of model development is mandatory to evaluate their effectiveness and strength critically, to allow the independent replication of the results, to appreciate the clinical relevance and finally to implement these models in daily practice [49,89]. Overall, as emerged from the adherence rates to the TRIPOD, the quality of the reporting of radiomics and AI studies is still not optimal for their introduction into clinical practice. The mean TRIPOD adherence rate was 62.9%, being higher than 50% in just over half (*n* = 10) of the 18 “high-quality papers”. Our findings largely confirmed those reported by Park et al. [49] in assessing the quality of the reporting of radiomics studies in oncology. We found a lower overall variability in the adherence rate to TRIPOD than the one they reported (range 53–73% versus 33–78%), but the means are comparable (62.9% versus 57.8%). The discrepancies between our findings and those previously reported are most likely related to the selection in our analysis of “high-quality papers” focused on such a specific topic. Moreover, the application of a radiomics-adapted TRIPOD statement to CNN-based approaches could influence the final results. Nonetheless, the proper use of artificial intelligence approaches in healthcare is mandatory. Similarly, to “classical” approaches, the high standards for model development, training, and testing are recommended, being essential requirements for the reliability and interpretability of the results. Accordingly, the quality assessment of AI studies should be ensured [90]. It should be acknowledged that some items of the TRIPOD checklist are unfit for AI investigations (as for radiomics [49]), and they should be somehow adapted or ignored. Indeed, an initiative to develop an “ad hoc” TRIPOD statement has been proposed [91]. However, we experienced that the majority of the TRIPOD’s domains already adapted for radiomics might be easily and successfully addressed, making AI studies more interpretable, transparent, reproducible and informative.

Many efforts have been made to standardize methodologies in advanced image analysis studies and to increase the reproducibility and generalizability of the obtained results [92]. Strict adherence to existing guidelines and prospective studies with the multicenter validation of predictive models are believed to be the prerequisites towards their clinical acceptance [37,52].

Additionally, to prove the reproducibility, robustness and reusability of the research results, data sharing should be embraced by the authors according to the four foundational principles—findability, accessibility, interoperability and reusability (FAIR). All components of the research process must be made FAIR, which is nowadays possible thanks to the emergence of numerous data repositories [93]. Data and methods sharing will contribute to extracting maximum benefits from research investments and help the radiomic, radiogenomic and AI fields to gain reputation.

Moreover, the trial phase assignment, which was done by applying a transfer learning strategy from the drug development process to imaging-derived biomarkers studies [52], failed in identifying the most promising models. Overall, the “high-quality papers” were rated as phases I or II, proving an immaturity of the investigations and suggesting that no preferred model can be recommended for future investigations.

Another strategy towards the non-invasive detection of predictive biomarkers in NSCLC is represented by liquid biopsy, which is a diagnostic tool that uses body fluids for biological testing. In the case of advanced NSCLC, the most promising type of liquid biopsy method is based on the isolation of circulating tumor DNA (ctDNA) from plasma samples [94]. Liquid biopsy has multiple advantages over traditional pathological testing, including relatively low costs, the potential to assess tumor heterogeneity, non-invasiveness and repeatability [95]. It is successfully used to guide treatment decisions in patients with actionable mutations [96,97,98]. The analysis of ctDNA is currently recommended at the time of diagnosis, particularly when cytological or tissue biopsy specimens are not adequate or cannot be obtained. However, liquid biopsy is still far from replacing tissue sampling. This consideration is mainly due to the risk of false-negative results. Indeed, the improvement of the analytic methods is necessary to increase sensitivity [99]. At present, there is no indication suggesting to perform liquid biopsy on select patients for immunotherapy, even if it is expected to be a promising method in this setting [100].

Our systematic review presents some limitations that should be acknowledged. Firstly, it was not registered in PROSPERO as recommended in the PRISMA statement; nonetheless, PROSPERO has recently changed rules for registration, accepting only reviews provided that data extraction has not started yet. Secondly, we did not search for potentially relevant papers in the EMBASE and in the CENTRAL database, as instead recommended by the Cochrane Handbook for Systematic Reviews of Interventions [101]. However, both EMABSE and CENTRAL are focused on drug development research, being designed to support information managers/pharmacovigilance and to register controlled trials, respectively. Conversely, papers focused on imaging are expected to be included in PubMed/MEDLINE. The other mandatory sources for additional paper searching according to the Cochrane Handbook for Systematic Reviews of Interventions (i.e., reference lists and ClinicalTrials.gov) were checked.

## 5. Conclusions

Image-based prediction models are not expected to replace traditional molecular pathology testing shortly. Further prospective studies with strict adherence to existing guidelines and multicenter validation need to be performed. The role of image-derived biomarkers could be relevant when invasive procedures are contraindicated, or in case biological samples are inadequate for molecular testing. The complementary and possibly synergistic combination of imaging and liquid biopsy could be the key to providing an attractive diagnostic alternative to traditional molecular pathology profiling in the landscape of personalized NSCLC treatment.

## Figures and Tables

**Table 1:** Biomarkers in non-small cell lung cancer.

Target/Biomarker			Frequency [28,29,30,31,32,33]	Targeted Therapy/Immunotherapy Options
EGFR mutation				Erlotinib [6], gefitinib [7], afatinib [20], osimertinib [21], dacomitinib [8]
	Overall		10–20% ^1^, 40–50% ^2^	
		Exon 19 deletion	≃45%	
		Exon 21 L858R mutation	≃40%	
		Others	≃15%	
ALK rearrangement			3–7%	Crizotinib [16], alectinib [10], ceritinib [9], brigatinib [13], lorlatinib [11]
ROS1 rearrangement			1–4%	Crizotinib [17], entrectinib [19]
BRAF mutation			1–5%	Dabrafenib + trametinib [12]
Tumor cells PD-L1 expression				Nivolumab ^3^ [22,27], pembrolizumab ^3^ [23,25], atezolizumab ^4^ [24], durvalumab ^4^ [26]
	<1%		30–40%	
	1–49%		30–40%	
	≥50%		≃30%	
Evolving target/biomarker ^5^				
RET rearrangement			1–3%	
ERRB2 (HER2) mutation			2–4%	
KRAS mutation			15–30%	
MET amplification			3–4%	

^1^ non-Asians; ^2^ Asians; ^3^ PD-1 inhibitor; ^4^ PD-L1 inhibitor; ^5^ No targeted therapies have been approved yet for these known oncogenic driver mutations.

**Table 2:** Summary of the study characteristics of “high-quality” and all eligible articles.

Study Characteristic		“High-Quality” Papers (*n* = 18)	All Eligible Papers (*n* = 24)
Year of publication			
	2014–2017	2 (11%)	4 (17%)
	2018–2020	16 (89%)	20 (83%)
Number of patients			
	0–100	2 (11%)	3 (12.5%)
	100–300	6 (33%)	9 (37.5%)
	300–500	3 (17%)	4 (17%)
	>500	7 (39%)	8 (33%)
Study type			
	Prospective	0	0
	Retrospective	18 (100%)	24 (100%)
Imaging modality			
	CT	14 (74%)	18 (75%)
	^18^F-FDG PET/CT	4 (26%)	6 (25%)
Molecule(s) of interest ^1^			
	EGFR	15 ^2^	20
	ALK	2	2
	ROS1	1	1
	BRAF	0	0
	RET	1	1
	HER2	0	0
	KRAS	0	3
	MET	0	0
	PD-L1	2	2
Imaging predictors ^3^			
	Visual qualitative CT features	8	10
	Conventional PET parameters	2	3
	Radiomic features	16	20
	CNN-based approaches	4	4
Type of validation			
	Internal	5 (28%)	7 (29%)
	Split sample	12 (67%)	15 (63%)
	Geographic external validation	1 (5%)	2 (8%)

^1^ Some studies had more than one molecule of interest and were considered as separate; ^2^ Fourteen studies focused on the prediction of EGFR mutation, and one on the prediction of EGFR mutation subtypes; ^3^ Some studies investigated the predictive potential of more than one type of imaging features and were considered separately; CNN = convolutional neural network; CT = computed tomography; ^18^F-FDG = fluorine-18 fluorodeoxyglucose; PD-L1 = programmed cell death ligand 1; PET = positron emission tomography.

**Table 3:** Studies using radiomic models, alone or combined with clinical models, to predict THE EGFR status.

Study	N (% EGFR+)	Study Population	Imaging Modality	Method	Validation	Main Results T, V	TRIPOD
**TRIPOD Adherence Rate 70–79%**							
[54]	637 (54%)	Stage I–IV AC	CT	Radiomics	Split Sample	AUC = 0.71, 0.69	73%
Selected CT r=Radiomic Features: First-Order Features (Mean, Skewness), GLCM Features (Homogeneity, Contrast), GLRLM Features (RLNU)							
[59]	844 (56%)	Stage I–IV AC	CT	Radiomics	Split Sample	AUC = 0.70, 0.64	73%
Selected CT Radiomic Features: Not Reported							
[55]	104 (62%)	Stage I–IV AC	CT	Radiomics Radiomics + Clinical	Split Sample	AUC = 0.92, 0.84 AUC = 0.90, 0.89	70%
Selected CT Radiomic Features: GLCM Features (Cluster Prominence), GLDM Features (LGE, DNN), GLSZM Features (SZHGE, SZLGE), Wavelet Features Selected Clinicopathologic Features: Sex, Smoking, Vascular Infiltration, Histological Subtype							
**TRIPOD Adherence Rate 60–69%**							
[69]	404 (46%)	Stage I–IV NSCLC	CT	Radiomics	Split Sample	AUC = 0.76, 0.78	67%
Selected CT Radiomic Features: First-Order Features (Median, Entropy), GLCM Features (Homogeneity), GLRLM Features (RLNU)							
[62]	180 (48%)	Stage III–IV NSCLC	CT	Radiomics Radiomics + Clinical	Split Sample	AUC = 0.76, 0.77 AUC = 0.86, 0.87	67%
Selected CT Radiomic Features: First-Order Features (Range, Skewness), GLRLM Features (HGRE), Wavelet Features Selected Clinicopathologic Features: Sex, Smoking, Histological subtype							
[68]	80 (38%)	Stage II–III NSCLC	PET/CT	Radiomics	Cross Validation	AUC = 0.83	63%
Selected Radiomic Features: Not Reported							
[56]	467 (64%)	Early-Stage AC	CT	Radiomics Radiomics + Clinical	Split Sample	AUC = 0.83, 0.79 AUC = 0.83, 0.78	63%
Selected CT Radiomic Features: First-Order Features (Energy, Entropy, Total Energy, Range, Flatness, Maximum 2D Diameter Slice, Surface Area), First-Order Features from LBP2D image (Major Axis, Maximum 2D Diameter Column, Maximum 2D Diameter Row, Maximum 3D Diameter, Sphericity), First-Order Features from LBP3D image (90th Percentile, Variance), GLCM Features (Sum Entropy, Autocorrelation, Cluster Prominence), GLSZM Features (HGZE, ZSNU), GLRLM Features (RLNU, GLV, HGRE, RE, SRLGE), GLDM Features (GLNU, DE, LGE), Wavelet Features Selected Clinicopathologic Features: Age, Histologic Subtype							
[57]	503 (61%)	Stage I–IV AC	CT	Radiomics Radiomics + Clinical	Split Sample	AUC = NR, 0.80 AUC = NR, 0.83	63%
Selected CT Radiomic Features: Not Reported Selected Clinicopathologic Features: Sex, Smoking							
**TRIPOD Adherence Rate 50–59%**							
[64]	115 (56%)	Stage I–IV AC	PET/CT	Radiomics Radiomics + Clinical	Cross Validation	AUC = 0.81 AUC = 0.82	57%
Selected PET Radiomic Features: First-Order Features (Mean, Concavity), GLCM Features (Homogeneity, Energy, Entropy, Contrast, Correlation) Selected CT Radiomic Features: First-Order Features (Range, Mean) Selected Clinicopathologic Features: Age, Sex, Smoking, Stage, Lesion Location							
[60]	51 (45%)	Stage I–III AC	CT	Radiomics	Cross Validation	AUC = 0.83	57%
Selected CT Radiomic Features: First-Order Features (Entropy, Energy, Volume, Shape Index), Wavelet Features							
[70]	579 (53%) 37 (24%) ^1^	Stage I–IV AC	CT	Radiomics	Split Sample External	AUC = NR, 0.65 AUC = 0.69	53%
Selected CT Radiomic Features: Not Clear							
[58]	1010 (50%)	Stage I–IV AC	CT	Radiomics Radiomics + Clinical	Split Sample	AUC = NR, 0.74 AUC = NR, 0.76	53%
Selected CT Radiomic Features: Not Clear Selected Clinicopathologic Features: Sex, Smoking							

^1^ The model was developed and trained using a cohort of Asian patients and further validated selecting a cohort of 37 non-Asian patients from a public dataset.; AC = adenocarcinoma; AUC = area under the curve; CT = computed tomography; DE = Dependence Entropy; DNN = Dependence Non-Uniformity Normalized; GLCM = Gray Level Co-occurrence Matrix; GLDM = Gray Level Dependence Matrix; GLRLM = Gray Level Run Length Matrix; GLSZM = Gray Level Size Zone Matrix; GLV = Gray Level Variance; HGRE = High Gray-level Run Emphasis; LBP3D = three-dimensional local binary pattern; LGE = low grey level emphasis; N = number of patients; NR = not reported; NGLDM = Neighborhood Grey-Level Different Matrix; NSCLC = non-small cell lung cancer; PET = positron emission tomography; SRLGE = Short-run low gray-level emphasis; SZHGE = Short Zone High Gray-Level Emphasis; SZLGE = Short Zone Low Gray-Level Emphasis; T = training dataset; V = validation dataset; TRIPOD = TRIPOD overall adherence rate; ZSNU = Zone-Size Non-Uniformity.

**Table 4:** Studies using the visual qualitative CT features-based models, alone or combined with clinical models, to predict the EGFR status.

Study	N (% EGFR+)	Study Population	Imaging Modality	Method	Validation	Main Results T, V	TRIPOD
**TRIPOD Adherence Rate 70–79%**							
[59]	844 (56%)	Stage I–IV AC	CT	Visual Qualitative Image Analysis	Split Sample	AUC = 0.76, 0.64	73%
Selected Visual Qualitative CT Features: Pleural Attachment, Border Definition, Spiculation, Density, Air Bronchogram, Bubblelike Lucency, Enhancement Heterogeneity, Vascular Convergence, Thickened Adjacent Bronchovascular Bundles, Pleural Indentation, Emphysema, Peripheral Fibrosis, Lymphadenopathy, Size, Long-Axis Diameter, Short-Axis Diameter							
**TRIPOD Adherence Rate 60–69%**							
[55]	104 (62%)	Stage I–IV AC	CT	Visual Qualitative Image Analysis + Clinical	Split Sample	AUC = 0.78, 0.77	67%
Selected Visual Qualitative CT Features: Spiculation, Tumor Necrosis Selected Clinicopathologic Features: Sex, Age, Visceral Pleural Infiltration, Histological Subtype							
[69]	404 (46%)	Stage I–IV NSCLC	CT	Visual Qualitative Image Analysis + Clinical	Split Sample	AUC = 0.69, 0.62	67%
Selected Visual Qualitative CT Features: Density, Location Selected Clinicopathologic Features: Sex							
[68]	80 (38%)	Stage II–III NSCLC	CT	Visual Qualitative Image Analysis	Cross Validation	AUC = 0.73	63%
Selected Visual Qualitative CT Features: Lobulation, Spiculation, Emphysema, Pleural Indentation							

AC = adenocarcinoma; AUC = area under the curve; CT = computed tomography; N = number of patients; NSCLC = non-small cell lung cancer; T = training dataset; V = validation dataset; TRIPOD = TRIPOD overall adherence rate.

**Table 5:** Visual qualitative CT features most commonly associated with EGFR mutation in the selected studies.

Clinicopathologic Feature	% Studies Reporting Statistically Significant Association
Spiculation	75%
Absence of Emphysema	75%
Pleural Indentation	50%
Subsolid Nodule	50%

**Table 6:** Studies using convolutional neural network (CNN)-based approaches, alone or combined with clinical models, to predict the EGFR status.

Study	N (% EGFR+)	Study Population	Imaging Modality	Method	Validation	Main Results T, V	TRIPOD
**TRIPOD Adherence Rate 70–79%**							
[59]	844 (56%)	Stage I–IV AC	CT	CNN	Split Sample	AUC = 0.85, 0.81	73%
**TRIPOD Adherence Rate 50–59%**							
[70]	579 (53%) 37 (24%) ^1^	Stage I–IV AC	CT	CNN	Split Sample External	AUC = NR, 0.76 AUC = 0.75	53%
[61]	503 (61%)	Stage I–IV AC	CT	CNN CNN + Clinical	Split Sample	AUC = NR, 0.78 AUC = NR, 0.84	53%
Selected Clinicopathologic Features: Sex, Smoking							
[58]	1010 (50%)	Stage I–IV AC	CT	CNN CNN + Clinical	Split Sample	AUC = NR, 0.81 AUC = NR, 0.83	53%
Selected Clinicopathologic Features: Sex, Smoking							

^1^ The model was developed and trained using a cohort of Asian patients and further validated selecting a cohort of 37 non-Asian patients from a public dataset.; AC = adenocarcinoma; AUC = area under the curve; CNN = convolutional neural networks; CT = computed tomography; N = number of patients; NR = not reported; T = training dataset; V = validation dataset; TRIPOD = TRIPOD overall adherence rate.

**Table 7:** Studies using the combined models to predict the EGFR status.

Study	N (% EGFR+)	Study Population	Imaging Modality	Method	Validation	Main Results T, V	TRIPOD
**TRIPOD adherence rate 70–79%**							
[54]	637 (54%)	Stage I–IV AC	CT	Radiomics + Visual Qualitative Image Analysis + Clinical	Split Sample	AUC = 0.76, 0.73	73%
Selected CT Radiomic Features: First-Order Features (Mean, Skewness), GLCM Features (Homogeneity, Contrast), GLRLM Features (RLNU) Selected Visual Qualitative CT Features: Emphysema Selected Clinicopathologic Features: Sex							
[63]	248 (54%)	Stage I–IV AC	PET/CT	Radiomics + PET Parameters Radiomics + PET Parameters + Clinical	Split Sample	AUC = 0.79, 0.85 AUC = 0.86, 0.87	70%
Selected PET Radiomic Features: First-Order Features (Compacity), GLCM Features (Energy), GLSZM Features (SZE, ZP) Selected CT Radiomic Features: First-Order Features (Maximum, Sphericity), GLSZM Features (ZLNU), GLRLM Features (HGRE), NGLDM Features (Busyness) Selected PET Parameters: SUVpeak Selected Clinicopathologic Features: Sex, Smoking							
**TRIPOD adherence rate 60–69%**							
[69]	404 (46%)	Stage I–IV NSCLC	CT	Radiomics + Visual Qualitative Image Analysis + Clinical	Split Sample	AUC = 0.80, 0.82	67%
Selected CT Radiomic Features: First-Order Features (Median, Entropy), GLCM Features (Homogeneity), GLRLM Features (RLNU) Selected Visual Qualitative CT Features: Long-Axis Diameter, Location Selected Clinicopathologic Features: Sex							
[68]	80 (38%)	Stage II–III NSCLC	PET/CT	Radiomics + Visual Qualitative Image Analysis	Cross Validation	AUC = 0.95	63%
Selected CT Radiomic Features: Not Clear Selected Visual Qualitative CT Features: Lobulation, Spiculation, Emphysema, Pleural indentation							
**TRIPOD adherence rate 50–59%**							
[58]	1010 (50%)	Stage I–IV AC	CT	CNN + Radiomics CNN + Radiomics + Clinical	Split Sample	AUC = NR, 0.81 AUC = NR, 0.83	53%
Selected CT Radiomic Features: Not Clear Selected Clinicopathologic Features: Sex, Smoking							

AC = adenocarcinoma; AUC = area under the curve; CNN = convolutional neural networks; CT = computed tomography; GLCM = Gray Level Co-occurrence Matrix; GLRLM = Gray Level Run Length Matrix; N = number of patients; NR = not reported; NSCLC = non-small cell lung cancer; PET = positron emission tomography; SUV = standardized uptake value; SUVpeak = maximum average SUV within a 1-cm^3^ spherical volume; SZE = short zone emphasis; T = training dataset; V = validation dataset; TRIPOD = TRIPOD overall adherence rate; ZP = zone percentage.

**Table 8:** Clinicopathologic features most commonly associated to the EGFR mutation in the selected studies.

Clinicopathologic Feature	% Studies Reporting Statistically Significant Association
Female Sex	90%
Non-Smoking Status	70%

**Table 9:** Studies using radiomic models, alone or combined with clinical models, to predict the two most common EGFR mutation subtypes (exon 19 deletion and exon 21 L858R mutation).

Study	N (exon19del: L858R)	Study Population	Imaging Modality	Method	Validation	Main Results T, V	TRIPOD
[54]	320 (130:190)	Stage I–IV AC	CT	Radiomics Radiomics + Clinical	Split Sample	AUC = 0.68, 0.71 AUC = 0.69, 0.76	73%
Selected CT Radiomic Features: First-Order Features (Mean, Skewness, Standard Deviation), GLCM Features (Homogeneity, Correlation, Entropy, Contrast), GLSZM Features (GLNU), GLRLM Features (LRE, SRE, RLNU) Selected Clinicopathologic Features: Age							

AC = adenocarcinoma; AUC = area under the curve; CT = computed tomography; exon19del = Exon 19 deletion; L858R = Exon 21 L858R mutation; N = number of patients; T = training dataset; V = validation dataset; TRIPOD = TRIPOD overall adherence rate.

**Table 10:** Studies that aim at predicting the ALK status.

Study	N (% ALK+)	Study Population	Imaging Modality	Method	Validation	Main Results (T) (V)	TRIPOD
[71]	172 (27%)	Stage I–IV NSCLC	CT	Visual Qualitative Image Analysis + Clinical	Split Sample	SE, SP, ACC = 86%, 77%, 81% (T) 83%, 78%, 79% (V)	60%
Selected Visual Qualitative CT Features: Location, Pleural Effusion, Pleural Tail Sign Selected Clinicopathologic Features: Age							

ACC = accuracy; CT = computed tomography; N = number of patients; NSCLC = non-small cell lung cancer; SE = sensitivity; SP = specificity; T = training dataset; V = validation dataset; TRIPOD = TRIPOD overall adherence rate.

**Table 11:** Studies that aim at identifying the ALK/ROS1/RET fusion-positive tumors versus the ALK/ROS1/RET fusion-negative tumors.

Study	N (% Fusion-Positive)	Study Population	Imaging Modality	Method	Validation	Main Results (T) (V)	TRIPOD
[75]	537 (16%)	Stage I–IV AC	PET/CT	Radiomics + PET Parameters + Visual Qualitative Image Analysis + Clinical	Cross Validation	SE, SP, = NR (T) 73%, 70% (V)	60%
Selected CT Radiomic Features: First-Order Features (Kurtosis), GLCM Features (Inverse Variance) Selected PET parameters: SUV_max_ Selected Visual Qualitative CT Features: Density, Mass Selected Clinicopathologic Features: Age, Stage							

AC = adenocarcinoma; CT = computed tomography; N = number of patients; PET = positron emission tomography; SE = sensitivity; SP = specificity; SUV = standardized uptake value; T = training dataset; V = validation dataset; TRIPOD = TRIPOD overall adherence rate.

**Table 12:** Studies using radiomic models, alone or combined with clinical models, to predict the PD-L1 expression levels.

Study	N (% PD-L1 ≥1% /≥50%)	Study Population	Imaging Modality	Method	Validation	Main Results T, V	TRIPOD
**Prediction of PD-L1 expression level ≥1%**							
[77] ^1^	399 (66%)	Stage I–IV NSCLC	PET/CT	Radiomics	Split Sample	AUC = NR, 0.86 AUC = NR, 0.97	53%
Selected PET Radiomic Features: First-Order Features (Maximum 2D Diameter Slice, Interquartile Range), Wavelet Features Selected CT Radiomic Features: First-Order Features (Maximum), First-Order Features from LBP3D Image (10th Percentile), GLRLM Features (RLNU), Wavelet Features							
**Prediction of PD-L1 expression level ≥50%**							
[76]	153 (35%)	Stage IIIb–IV AC	CT	Radiomics + Clinical	Bootstrapping Validation	AUC = 0.67, 0.67	73%
Selected CT Radiomic Features: GLCM Features (Energy), GLRLM Features (RV, RE, SRHGE) Selected Clinicopathologic Features: Age, Sex, Smoking, EGFR status							
[77] ^1^	399 (21%)	Stage I-IV NSCLC	PET/CT	Radiomics	Split Sample	AUC = NR, 0.910 AUC = NR, 0.770	53%
Selected PET Radiomic Features: First-Order Features (Maximum 2D Diameter Slice, Interquartile Range), Wavelet Features Selected CT Radiomic Features: First-Order Features (Maximum), First-Order Features from LBP3D Image (10th Percentile), GLRLM Features (RLNU), Wavelet Features							

^1^ Two different PD-L1 test kits were used to measure the PD-L1 expression level in this study and the patients were divided into two groups taking into account this aspect; a common model was created and validated on the two cohorts separately. AC = adenocarcinoma; CT = computed tomography; N = number of patients; NR = not reported; NSCLC = non-small cell lung cancer; PET = positron emission tomography; RE = run entropy; RV = run variance; SRHGE = short-run high gray-level emphasis; T = training dataset; V = validation dataset; TRIPOD = TRIPOD overall adherence rate.

## Supplementary Materials

The following are available online at https://www.mdpi.com/2075-4418/10/6/359/s1, Search strategy; Figure S1: Flow chart of the literature selection process; Figure S2: QUADAS-2 tool for quality assessment of each included study; Table S1: Details of molecular genetic alterations or PD-L1 expression stratified according to the stage (early versus advanced) in the “high-quality” papers; Table S2: Summary of the study reporting AUC and 95% confidence interval to predict genetic alterations and immunotherapy targets.

## References

[1] Bray F., Ferlay J., Soerjomataram I., Siegel R.L., Torre L.A., Jemal A. (2018). Global cancer statistics 2018: GLOBOCAN estimates of incidence and mortality worldwide for 36 cancers in 185 countries. CA Cancer J. Clin..

[2] Remon J., Ahn M.-J., Girard N., Johnson M., Kim D.-W., Lopes G., Pillai R.N., Solomon B., Villacampa G., Zhou Q. (2019). Advanced-Stage Non–Small Cell Lung Cancer: Advances in Thoracic Oncology 2018. J. Thorac. Oncol..

[3] Lindeman N.I., Cagle P.T., Aisner D.L., Arcila M.E., Beasley M.B., Bernicker E.H., Colasacco C., Dacic S., Hirsch F.R., Kerr K. (2018). Updated Molecular Testing Guideline for the Selection of Lung Cancer Patients for Treatment With Targeted Tyrosine Kinase Inhibitors: Guideline From the College of American Pathologists, the International Association for the Study of Lung Cancer, and the Association for Molecular Pathology. Arch. Pathol. Lab. Med..

[4] Kalemkerian G.P., Narula N., Kennedy E.B., Biermann W.A., Donington J., Leighl N.B., Lew M., Pantelas J., Ramalingam S.S., Reck M. (2018). Molecular Testing Guideline for the Selection of Patients With Lung Cancer for Treatment With Targeted Tyrosine Kinase Inhibitors: American Society of Clinical Oncology Endorsement of the College of American Pathologists/International Association for the Study of Lung Cancer/Association for Molecular Pathology Clinical Practice Guideline Update. J. Clin. Oncol..

[5] Planchard D., Popat S., Kerr K., Novello S., Smit E.F., Faivre-Finn C., Mok T.S., Reck M., Van Schil P.E., Hellmann M.D. (2018). Metastatic non-small cell lung cancer: ESMO Clinical Practice Guidelines for diagnosis, treatment and follow-up. Ann. Oncol..

[6] Shepherd F.A., Rodrigues Pereira J., Ciuleanu T., Tan E.H., Hirsh V., Thongprasert S., Campos D., Maoleekoonpiroj S., Smylie M., Martins R. (2005). Erlotinib in Previously Treated Non–Small-Cell Lung Cancer. N. Engl. J. Med..

[7] Mok T.S., Wu Y.-L., Thongprasert S., Yang C.-H., Chu D.-T., Saijo N., Sunpaweravong P., Han B., Margono B., Ichinose Y. (2009). Gefitinib or Carboplatin–Paclitaxel in Pulmonary Adenocarcinoma. N. Engl. J. Med..

[8] Wu Y.-L., Cheng Y., Zhou X., Lee K.H., Nakagawa K., Niho S., Tsuji F., Linke R., Rosell R., Corral J. (2017). Dacomitinib versus gefitinib as first-line treatment for patients with EGFR-mutation-positive non-small-cell lung cancer (ARCHER 1050): A randomised, open-label, phase 3 trial. Lancet Oncol..

[9] Soria J.-C., Tan D.S.W., Chiari R., Wu Y.-L., Paz-Ares L., Wolf J., Geater S.L., Orlov S., Cortinovis D., Yu C.-J. (2017). First-line ceritinib versus platinum-based chemotherapy in advanced ALK -rearranged non-small-cell lung cancer (ASCEND-4): A randomised, open-label, phase 3 study. Lancet.

[10] Hida T., Nokihara H., Kondo M., Kim Y.H., Azuma K., Seto T., Takiguchi Y., Nishio M., Yoshioka H., Imamura F. (2017). Alectinib versus crizotinib in patients with ALK -positive non-small-cell lung cancer (J-ALEX): An open-label, randomised phase 3 trial. Lancet.

[11] Shaw A.T., Solomon B.J., Besse B., Bauer T.M., Lin C.-C., Soo R.A., Riely G.J., Ou S.-H.I., Clancy J.S., Li S. (2019). ALK Resistance Mutations and Efficacy of Lorlatinib in Advanced Anaplastic Lymphoma Kinase-Positive Non–Small-Cell Lung Cancer. J. Clin. Oncol..

[12] Gautschi O., Milia J., Cabarrou B., Bluthgen M.-V., Besse B., Smit E.F., Wolf J., Peters S., Früh M., Koeberle D. (2015). Targeted Therapy for Patients with BRAF-Mutant Lung Cancer Results from the European EURAF Cohort. J. Thorac. Oncol..

[13] Camidge D.R., Kim H.R., Ahn M.-J., Yang J.C.-H., Han J.-Y., Lee J.-S., Hochmair M.J., Li J.Y.-C., Chang G.-C., Lee K.H. (2018). Brigatinib versus Crizotinib in ALK -Positive Non–Small-Cell Lung Cancer. N. Engl. J. Med..

[14] Han J.-Y., Park K., Kim S.-W., Lee D.H., Kim H.Y., Kim H.T., Ahn M.J., Yun T., Ahn J.S., Suh C. (2012). First-SIGNAL: First-Line Single-Agent Iressa Versus Gemcitabine and Cisplatin Trial in Never-Smokers With Adenocarcinoma of the Lung. J. Clin. Oncol..

[15] Maemondo M., Inoue A., Kobayashi K., Sugawara S., Oizumi S., Isobe H., Gemma A., Harada M., Yoshizawa H., Kinoshita I. (2010). Gefitinib or Chemotherapy for Non–Small-Cell Lung Cancer with Mutated EGFR. N. Engl. J. Med..

[16] Solomon B.J., Mok T., Kim D.-W., Wu Y.-L., Nakagawa K., Mekhail T., Felip E., Cappuzzo F., Paolini J., Usari T. (2014). First-Line Crizotinib versus Chemotherapy in ALK -Positive Lung Cancer. N. Engl. J. Med..

[17] Shaw A.T., Ou S.-H.I., Bang Y.-J., Camidge D.R., Solomon B.J., Salgia R., Riely G.J., Varella-Garcia M., Shapiro G.I., Costa D.B. (2014). Crizotinib in ROS1 -Rearranged Non–Small-Cell Lung Cancer. N. Engl. J. Med..

[18] Mazières J., Zalcman G., Crinò L., Biondani P., Barlesi F., Filleron T., Dingemans A.-M.C., Léna H., Monnet I., Rothschild S.I. (2015). Crizotinib Therapy for Advanced Lung Adenocarcinoma and a ROS1 Rearrangement: Results From the EUROS1 Cohort. J. Clin. Oncol..

[19] Drilon A., Siena S., Dziadziuszko R., Barlesi F., Krebs M.G., Shaw A.T., de Braud F., Rolfo C., Ahn M.-J., Wolf J. (2020). Entrectinib in ROS1 fusion-positive non-small-cell lung cancer: Integrated analysis of three phase 1–2 trials. Lancet Oncol..

[20] Sequist L.V., Yang J.C.-H., Yamamoto N., O’Byrne K., Hirsh V., Mok T., Geater S.L., Orlov S., Tsai C.-M., Boyer M. (2013). Phase III Study of Afatinib or Cisplatin Plus Pemetrexed in Patients With Metastatic Lung Adenocarcinoma With EGFR Mutations. J. Clin. Oncol..

[21] Soria J.-C., Ohe Y., Vansteenkiste J., Reungwetwattana T., Chewaskulyong B., Lee K.H., Dechaphunkul A., Imamura F., Nogami N., Kurata T. (2018). Osimertinib in Untreated EGFR -Mutated Advanced Non–Small-Cell Lung Cancer. N. Engl. J. Med..

[22] Carbone D.P., Reck M., Paz-Ares L., Creelan B., Horn L., Steins M., Felip E., van den Heuvel M.M., Ciuleanu T.-E., Badin F. (2017). First-Line Nivolumab in Stage IV or Recurrent Non–Small-Cell Lung Cancer. N. Engl. J. Med..

[23] Mok T.S.K., Wu Y., Kudaba I., Kowalski D.M., Cho B.C., Turna H.Z., Castro G., Srimuninnimit V., Laktionov K.K., Bondarenko I. (2019). Pembrolizumab versus chemotherapy for previously untreated, PD-L1-expressing, locally advanced or metastatic non-small-cell lung cancer (KEYNOTE-042): A randomised, open-label, controlled, phase 3 trial. Lancet.

[24] Socinski M.A., Jotte R.M., Cappuzzo F., Orlandi F., Stroyakovskiy D., Nogami N., Rodríguez-Abreu D., Moro-Sibilot D., Thomas C.A., Barlesi F. (2018). Atezolizumab for First-Line Treatment of Metastatic Nonsquamous NSCLC. N. Engl. J. Med..

[25] Garon E.B., Rizvi N.A., Hui R., Leighl N., Balmanoukian A.S., Eder J.P., Patnaik A., Aggarwal C., Gubens M., Horn L. (2015). Pembrolizumab for the Treatment of Non–Small-Cell Lung Cancer. N. Engl. J. Med..

[26] Murakami S. (2019). Durvalumab for the treatment of non-small cell lung cancer. Expert Rev. Anticancer Ther..

[27] Rizvi N.A., Mazières J., Planchard D., Stinchcombe T.E., Dy G.K., Antonia S.J., Horn L., Lena H., Minenza E., Mennecier B. (2015). Activity and safety of nivolumab, an anti-PD-1 immune checkpoint inhibitor, for patients with advanced, refractory squamous non-small-cell lung cancer (CheckMate 063): A phase 2, single-arm trial. Lancet Oncol..

[28] Zhang Y.L., Yuan J.Q., Wang K.F., Fu X.H., Han X.R., Threapleton D., Yang Z.Y., Mao C., Tang J.L. (2016). The prevalence of EGFR mutation in patients with non-small cell lung cancer: A systematic review and meta-analysis. Oncotarget.

[29] Sweeney S.M., Cerami E., Baras A., Pugh T.J., Schultz N., Stricker T., Lindsay J., Del Vecchio Fitz C., Kumari P., Micheel C. (2017). AACR Project GENIE: Powering Precision Medicine through an International Consortium. Cancer Discov..

[30] Skov B.G., Rørvig S.B., Jensen T.H.L., Skov T. (2020). The prevalence of programmed death ligand-1 (PD-L1) expression in non-small cell lung cancer in an unselected, consecutive population. Mod. Pathol..

[31] Sholl L.M., Aisner D.L., Varella-Garcia M., Berry L.D., Dias-Santagata D., Wistuba I.I., Chen H., Fujimoto J., Kugler K., Franklin W.A. (2015). Multi-institutional oncogenic driver mutation analysis in lung adenocarcinoma: The lung cancer mutation consortium experience. J. Thorac. Oncol..

[32] Barlesi F., Mazieres J., Merlio J.-P., Debieuvre D., Mosser J., Lena H., Ouafik L., Besse B., Rouquette I., Westeel V. (2016). Routine molecular profiling of patients with advanced non-small-cell lung cancer: Results of a 1-year nationwide programme of the French Cooperative Thoracic Intergroup (IFCT). Lancet.

[33] Midha A., Dearden S., McCormack R. (2015). EGFR mutation incidence in non-Small-cell lung cancer of adenocarcinoma histology: A systematic review and global map by ethnicity (mutMapII). Am. J. Cancer Res..

[34] Vansteenkiste J., Crino L., Dooms C., Douillard J.Y., Faivre-Finn C., Lim E., Rocco G., Senan S., Van Schil P., Veronesi G. (2014). 2nd ESMO Consensus Conference on Lung Cancer: Early-stage non-small-cell lung cancer consensus on diagnosis, treatment and follow-up. Ann. Oncol..

[35] Litjens G., Kooi T., Bejnordi B.E., Setio A.A.A., Ciompi F., Ghafoorian M., van der Laak J.A.W.M., van Ginneken B., Sánchez C.I. (2017). A survey on deep learning in medical image analysis. Med. Image Anal..

[36] Gillies R.J., Kinahan P.E., Hricak H. (2016). Radiomics: Images Are More than Pictures, They Are Data. Radiology.

[37] deSouza N.M., Achten E., Alberich-Bayarri A., Bamberg F., Boellaard R., Clément O., Fournier L., Gallagher F., Golay X., Heussel C.P. (2019). Validated imaging biomarkers as decision-making tools in clinical trials and routine practice: Current status and recommendations from the EIBALL* subcommittee of the European Society of Radiology (ESR). Insights Imaging.

[38] Rutman A.M., Kuo M.D. (2009). Radiogenomics: Creating a link between molecular diagnostics and diagnostic imaging. Eur. J. Radiol..

[39] Bodalal Z., Trebeschi S., Nguyen-Kim T.D.L., Schats W., Beets-Tan R. (2019). Radiogenomics: Bridging imaging and genomics. Abdom. Radiol..

[40] Chartrand G., Cheng P.M., Vorontsov E., Drozdzal M., Turcotte S., Pal C.J., Kadoury S., Tang A. (2017). Deep Learning: A Primer for Radiologists. RadioGraphics.

[41] Neri E., Del Re M., Paiar F., Erba P., Cocuzza P., Regge D., Danesi R. (2018). Radiomics and liquid biopsy in oncology: The holons of systems medicine. Insights Imaging.

[42] Sollini M., Bandera F., Kirienko M. (2019). Quantitative imaging biomarkers in nuclear medicine: From SUV to image mining studies. Highlights from annals of nuclear medicine 2018. Eur. J. Nucl. Med. Mol. Imaging.

[43] Park H., Sholl L.M., Hatabu H., Awad M.M., Nishino M. (2019). Imaging of precision therapy for lung cancer: Current state of the art. Radiology.

[44] Sollini M., Cozzi L., Ninatti G., Antunovic L., Cavinato L., Chiti A., Kirienko M. (2020). PET/CT radiomics in breast cancer: Mind the step. Methods.

[45] Sollini M., Cozzi L., Antunovic L., Chiti A., Kirienko M. (2017). PET Radiomics in NSCLC: State of the art and a proposal for harmonization of methodology. Sci. Rep..

[46] Home-ClinicalTrials.gov. https://clinicaltrials.gov/ct2/home.

[47] Whiting P., Rutjes A., Westwood M., Mallett S., Deeks J., Reitsma J., Leeflang M., Sterne J., Bossuyt P., Rutjes A. (2011). QUADAS-2: A revised tool for the quality assessment of diagnostic accuracy studies. Ann. Intern. Med..

[48] Collins G.S., Reitsma J.B., Altman D.G., Moons K.G.M. (2015). Transparent reporting of a multivariable prediction model for individual prognosis or diagnosis (TRIPOD): The TRIPOD statement. Ann. Intern. Med..

[49] Park J.E., Kim D., Kim H.S., Park S.Y., Kim J.Y., Cho S.J., Shin J.H., Kim J.H. (2020). Quality of science and reporting of radiomics in oncologic studies: Room for improvement according to radiomics quality score and TRIPOD statement. Eur. Radiol..

[50] Mandrekar J.N. (2010). Receiver Operating Characteristic Curve in Diagnostic Test Assessment. J. Thorac. Oncol..

[51] Fawcett T. (2006). An introduction to ROC analysis. Pattern Recognit. Lett..

[52] Sollini M., Antunovic L., Chiti A., Kirienko M. (2019). Towards clinical application of image mining: A systematic review on artificial intelligence and radiomics. Eur. J. Nucl. Med. Mol. Imaging.

[53] Sollini M., Gelardi F., Matassa G., Delgado Bolton R.C., Chiti A., Kirienko M. (2020). Interdisciplinarity: An essential requirement for translation of radiomics research into clinical practice–a systematic review focused on thoracic oncology. Rev. Española Med. Nucl. e Imagen Mol. (English Ed.).

[54] Zhao W., Wu Y., Xu Y., Sun Y., Gao P., Tan M., Ma W., Li C., Jin L., Hua Y. (2020). The Potential of Radiomics Nomogram in Non-invasively Prediction of Epidermal Growth Factor Receptor Mutation Status and Subtypes in Lung Adenocarcinoma. Front. Oncol..

[55] Lu X., Li M., Zhang H.-M., Hua S., Meng F., Yang H., Li X., Cao D. (2020). A novel radiomic nomogram for predicting epidermal growth factor receptor mutation in peripheral lung adenocarcinoma. Phys. Med. Biol..

[56] Yang X., Dong X., Wang J., Li W., Gu Z., Gao D., Zhong N., Guan Y. (2019). Computed Tomography-Based Radiomics Signature: A Potential Indicator of Epidermal Growth Factor Receptor Mutation in Pulmonary Adenocarcinoma Appearing as a Subsolid Nodule. Oncologist.

[57] Jia T.Y., Xiong J.F., Li X.Y., Yu W., Xu Z.Y., Cai X.W., Ma J.C., Ren Y.C., Larsson R., Zhang J. (2019). Identifying EGFR mutations in lung adenocarcinoma by noninvasive imaging using radiomics features and random forest modeling. Eur. Radiol..

[58] Li X.Y., Xiong J.F., Jia T.Y., Shen T.L., Hou R.P., Zhao J., Fu X.L. (2018). Detection of epithelial growth factor receptor (EGFR) mutations on CT images of patients with lung adenocarcinoma using radiomics and/or multi-level residual convolutionary neural networks. J. Thorac. Dis..

[59] Wang S., Shi J., Ye Z., Dong D., Yu D., Zhou M., Liu Y., Gevaert O., Wang K., Zhu Y. (2019). Predicting EGFR mutation status in lung adenocarcinoma on computed tomography image using deep learning. Eur. Respir. J..

[60] Li Y., Lu L., Xiao M., Dercle L., Huang Y., Zhang Z., Schwartz L.H., Li D., Zhao B. (2018). CT Slice Thickness and Convolution Kernel Affect Performance of a Radiomic Model for Predicting EGFR Status in Non-Small Cell Lung Cancer: A Preliminary Study. Sci. Rep..

[61] Xiong J.-F., Jia T.-Y., Li X.-Y., Yu W., Xu Z.-Y., Cai X.-W., Fu L., Zhang J., Qin B.-J., Fu X.-L. (2018). Identifying epidermal growth factor receptor mutation status in patients with lung adenocarcinoma by three-dimensional convolutional neural networks. Br. J. Radiol..

[62] Zhang L., Chen B., Liu X., Song J., Fang M., Hu C., Dong D., Li W., Tian J. (2018). Quantitative Biomarkers for Prediction of Epidermal Growth Factor Receptor Mutation in Non-Small Cell Lung Cancer. Transl. Oncol..

[63] Zhang J., Zhao X., Zhao Y., Zhang J., Zhang Z., Wang J., Wang Y., Dai M., Han J. (2020). Value of pre-therapy 18F-FDG PET/CT radiomics in predicting EGFR mutation status in patients with non-small cell lung cancer. Eur. J. Nucl. Med. Mol. Imaging.

[64] Li X., Yin G., Zhang Y., Dai D., Liu J., Chen P., Zhu L., Ma W., Xu W. (2019). Predictive Power of a Radiomic Signature Based on 18F-FDG PET/CT Images for EGFR Mutational Status in NSCLC. Front. Oncol..

[65] Koyasu S., Nishio M., Isoda H., Nakamoto Y., Togashi K. (2020). Usefulness of gradient tree boosting for predicting histological subtype and EGFR mutation status of non-small cell lung cancer on 18F FDG-PET/CT. Ann. Nucl. Med..

[66] Wang X., Kong C., Xu W., Yang S., Shi D., Zhang J., Du M., Wang S., Bai Y., Zhang T. (2019). Decoding tumor mutation burden and driver mutations in early stage lung adenocarcinoma using CT-based radiomics signature. Thorac. Cancer.

[67] Li S., Ding C., Zhang H., Song J., Wu L. (2019). Radiomics for the prediction of EGFR mutation subtypes in non-small cell lung cancer. Med. Phys..

[68] Jiang M., Zhang Y., Xu J., Ji M., Guo Y., Guo Y., Xiao J., Yao X., Shi H., Zeng M. (2019). Assessing EGFR gene mutation status in non-small cell lung cancer with imaging features from PET/CT. Nucl. Med. Commun..

[69] Tu W., Sun G., Fan L., Wang Y., Xia Y., Guan Y., Li Q., Zhang D., Liu S., Li Z. (2019). Radiomics signature: A potential and incremental predictor for EGFR mutation status in NSCLC patients, comparison with CT morphology. Lung Cancer.

[70] Zhao W., Yang J., Ni B., Bi D., Sun Y., Xu M., Zhu X., Li C., Jin L., Gao P. (2019). Toward automatic prediction of EGFR mutation status in pulmonary adenocarcinoma with 3D deep learning. Cancer Med..

[71] Yamamoto S., Korn R.L., Oklu R., Migdal C., Gotway M.B., Weiss G.J., Iafrate A.J., Kim D.W., Kuo M.D. (2014). ALK molecular phenotype in non-small cell lung cancer: CT radiogenomic characterization. Radiology.

[72] Rizzo S., Raimondi S., de Jong E.E.C., van Elmpt W., De Piano F., Petrella F., Bagnardi V., Jochems A., Bellomi M., Dingemans A.M. (2019). Genomics of non-small cell lung cancer (NSCLC): Association between CT-based imaging features and EGFR and K-RAS mutations in 122 patients—An external validation. Eur. J. Radiol..

[73] Rios Velazquez E., Parmar C., Liu Y., Coroller T.P., Cruz G., Stringfield O., Ye Z., Makrigiorgos M., Fennessy F., Mak R.H. (2017). Somatic Mutations Drive Distinct Imaging Phenotypes in Lung Cancer. Cancer Res..

[74] Gevaert O., Echegaray S., Khuong A., Hoang C.D., Shrager J.B., Jensen K.C., Berry G.J., Guo H.H., Lau C., Plevritis S.K. (2017). Predictive radiogenomics modeling of EGFR mutation status in lung cancer. Sci. Rep..

[75] Yoon H.J., Sohn I., Cho J.H., Lee H.Y., Kim J.H., Choi Y.L., Kim H., Lee G., Lee K.S., Kim J. (2015). Decoding tumor phenotypes for ALK, ROS1, and RET fusions in lung adenocarcinoma using a radiomics approach. Medicine.

[76] Yoon J., Suh Y.J., Han K., Cho H., Lee H., Hur J., Choi B.W. (2020). Utility of CT radiomics for prediction of PD-L1 expression in advanced lung adenocarcinomas. Thorac. Cancer.

[77] Jiang M., Sun D., Guo Y., Guo Y., Xiao J., Wang L., Yao X. (2020). Assessing PD-L1 Expression Level by Radiomic Features From PET/CT in Nonsmall Cell Lung Cancer Patients: An Initial Result. Acad. Radiol..

[78] Jansen R.W., van Amstel P., Martens R.M., Kooi I.E., Wesseling P., de Langen A.J., Menke-Van der Houven van Oordt C.W., Jansen B.H.E., Moll A.C., Dorsman J.C. (2018). Non-invasive tumor genotyping using radiogenomic biomarkers, a systematic review and oncology-wide pathway analysis. Oncotarget.

[79] Mendoza D.P., Stowell J., Muzikansky A., Shepard J.-A.O., Shaw A.T., Digumarthy S.R. (2019). Computed Tomography Imaging Characteristics of Non–Small-Cell Lung Cancer With Anaplastic Lymphoma Kinase Rearrangements: A Systematic Review and Meta-Analysis. Clin. Lung Cancer.

[80] Sanduleanu S., Woodruff H.C., de Jong E.E.C., van Timmeren J.E., Jochems A., Dubois L., Lambin P. (2018). Tracking tumor biology with radiomics: A systematic review utilizing a radiomics quality score. Radiother. Oncol..

[81] Shi Y., Au J.S.-K., Thongprasert S., Srinivasan S., Tsai C.-M., Khoa M.T., Heeroma K., Itoh Y., Cornelio G., Yang P.-C. (2014). A Prospective, Molecular Epidemiology Study of EGFR Mutations in Asian Patients with Advanced Non–Small-Cell Lung Cancer of Adenocarcinoma Histology (PIONEER). J. Thorac. Oncol..

[82] Dearden S., Stevens J., Wu Y.-L., Blowers D. (2013). Mutation incidence and coincidence in non small-cell lung cancer: Meta-analyses by ethnicity and histology (mutMap). Ann. Oncol..

[83] Park S.H., Han K. (2018). Methodologic Guide for Evaluating Clinical Performance and Effect of Artificial Intelligence Technology for Medical Diagnosis and Prediction. Radiology.

[84] Dogan S., Shen R., Ang D.C., Johnson M.L., D’Angelo S.P., Paik P.K., Brzostowski E.B., Riely G.J., Kris M.G., Zakowski M.F. (2012). Molecular Epidemiology of EGFR and KRAS Mutations in 3026 Lung Adenocarcinomas: Higher Susceptibility of Women to Smoking-Related KRAS-Mutant Cancers. Clin. Cancer Res..

[85] Teixidó C., Vilariño N., Reyes R., Reguart N. (2018). PD-L1 expression testing in non-small cell lung cancer. Ther. Adv. Med. Oncol..

[86] Haragan A., Field J.K., Davies M.P.A., Escriu C., Gruver A., Gosney J.R. (2019). Heterogeneity of PD-L1 expression in non-small cell lung cancer: Implications for specimen sampling in predicting treatment response. Lung Cancer.

[87] Patel S.P., Kurzrock R. (2015). PD-L1 Expression as a Predictive Biomarker in Cancer Immunotherapy. Mol. Cancer Ther..

[88] Whiting P.F., Rutjes A.W.S., Westwood M.E., Mallett S., Deeks J.J., Reitsma J.B., Leeflang M.M.G., Sterne J.A.C., Bossuyt P.M.M. (2016). QUADAS-2: Strumento per valutare la qualità degli studi di accuratezza diagnostica. Evidence.

[89] Heus P., Damen J.A.A.G., Pajouheshnia R., Scholten R.J.P.M., Reitsma J.B., Collins G.S., Altman D.G., Moons K.G.M., Hooft L. (2018). Poor reporting of multivariable prediction model studies: Towards a targeted implementation strategy of the TRIPOD statement. BMC Med..

[90] Nagendran M., Chen Y., Lovejoy C.A., Gordon A.C., Komorowski M., Harvey H., Topol E.J., Ioannidis J.P.A., Collins G.S., Maruthappu M. (2020). Artificial intelligence versus clinicians: Systematic review of design, reporting standards, and claims of deep learning studies. BMJ.

[91] Collins G.S., Moons K.G.M. (2019). Reporting of artificial intelligence prediction models. Lancet.

[92] Zwanenburg A., Vallières M., Abdalah M.A., Aerts H.J.W.L., Andrearczyk V., Apte A., Ashrafinia S., Bakas S., Beukinga R.J., Boellaard R. (2020). The Image Biomarker Standardization Initiative: Standardized Quantitative Radiomics for High-Throughput Image-based Phenotyping. Radiology.

[93] Wilkinson M.D., Dumontier M., Aalbersberg I.J., Appleton G., Axton M., Baak A., Blomberg N., Boiten J.-W., da Silva Santos L.B., Bourne P.E. (2016). The FAIR Guiding Principles for scientific data management and stewardship. Sci. Data.

[94] Rolfo C., Mack P.C., Scagliotti G.V., Baas P., Barlesi F., Bivona T.G., Herbst R.S., Mok T.S., Peled N., Pirker R. (2018). Liquid Biopsy for Advanced Non-Small Cell Lung Cancer (NSCLC): A Statement Paper from the IASLC. J. Thorac. Oncol..

[95] Goldman J.W., Noor Z.S., Remon J., Besse B., Rosenfeld N. (2018). Are liquid biopsies a surrogate for tissue EGFR testing?. Ann. Oncol..

[96] Mayo-de-las-Casas C., Jordana-Ariza N., Garzón-Ibañez M., Balada-Bel A., Bertrán-Alamillo J., Viteri-Ramírez S., Reguart N., Muñoz-Quintana M.A., Lianes-Barragan P., Camps C. (2017). Large scale, prospective screening of EGFR mutations in the blood of advanced NSCLC patients to guide treatment decisions. Ann. Oncol..

[97] Laufer-Geva S., Rozenblum A.B., Twito T., Grinberg R., Dvir A., Soussan-Gutman L., Ilouze M., Roisman L.C., Dudnik E., Zer A. (2018). The Clinical Impact of Comprehensive Genomic Testing of Circulating Cell-Free DNA in Advanced Lung Cancer. J. Thorac. Oncol..

[98] Remon J., Caramella C., Jovelet C., Lacroix L., Lawson A., Smalley S., Howarth K., Gale D., Green E., Plagnol V. (2017). Osimertinib benefit inEGFR-mutant NSCLC patients withT790M-mutation detected by circulating tumour DNA. Ann. Oncol..

[99] Saarenheimo J., Eigeliene N., Andersen H., Tiirola M., Jekunen A. (2019). The value of liquid biopsies for guiding therapy decisions in non-small cell lung cancer. Front. Oncol..

[100] Hofman P., Heeke S., Alix-Panabières C., Pantel K. (2019). Liquid biopsy in the era of immuno-oncology: Is it ready for prime-time use for cancer patients?. Ann. Oncol..

[101] Higgins J.P.T., Thomas J., Chandler J., Cumpston M., Li T., Page M.J., Welch V.A. (2019). Cochrane Handbook for Systematic Reviews of Interventions.

