# Supplementary material for: Imaging-Based Prediction of Molecular Therapy Targets in NSCLC by Radiogenomics and AI Approaches: A Systematic Review
Source: Diagnostics (Basel). 2020 May 30;10(6):359. doi: 10.3390/diagnostics10060359 (PMC7345054; doi:10.3390/diagnostics10060359)
Supplement: Supplementary file 1 [file diagnostics-10-00359-s001.pdf]

Supplementary Material

Imaging-Based Prediction of Molecular Therapy Targets in NSCLC by Radiogenomics and AI Approaches: A Systematic Review

Gaia Ninatti [1], Margarita Kirienko [2], Emanuele Neri [3], Martina Sollini [1,4,*] and Arturo Chiti [1,4]

[1] Humanitas University, Pieve Emanuele (Milan), Italy
[2] Fondazione IRCCS Istituto Nazionale dei Tumori, Milan, Italy
[3] Department of Translational Research, Diagnostic Radiology 3, University of Pisa, Italy
[4] Humanitas Clinical and Research Center - IRCCS, Rozzano (Milan), Italy
* Correspondence: martina.sollini@cancercenter.humanitas.it

Search strategy

Radiogenomics (A)
("radiogenomics" OR "radiogenomic" OR "imaging genomic" OR "imaging genomics")

Lung cancer (B)
("lung" OR "NSCLC" OR "pulmonary")

Molecular alterations/targeted therapy/PD-1 and PD-L1/immunotherapy (C)
("genomic" OR “genomics” OR “gene” OR “genes” OR “genetic” OR “genetics” OR “metagene” OR “metagenes” OR “mutation” OR “mutations” OR "RNA” OR “miRNA" OR "mRNA" OR "ALK" OR "EGFR" OR "KRAS" OR "RET" OR "ROS1" OR "VEGF" OR "MET" OR "BRAF" OR "ERBB2" OR "HER2" OR "PD-1" OR "PD-L1" OR "target" OR "targeted" OR "TKI" OR “molecular” OR “kinase” OR “monoclonal” OR “antibody” OR “immunotherapy”)

Imaging (D)
("texture" OR "textural" OR "radiomic" OR "radiomics" OR “imaging features” OR “imaging biomarker” OR “imaging biomarkers” OR “imaging characteristics” OR “deep learning” OR “CNN” OR “convolutional”)

PubMed/MEDLINE Search

1. (A) AND (B):
   ("radiogenomics" OR "radiogenomic" OR "imaging genomic" OR "imaging genomics") AND ("lung" OR "NSCLC" OR "pulmonary")

2. ((C) AND (D)) AND (B)

(("genomic" OR “genomics” OR “gene” OR “genes” OR “genetic” OR “genetics” OR “metagene” OR “metagenes” OR “mutation” OR “mutations” OR "RNA” OR “miRNA" OR "mRNA" OR "ALK" OR "EGFR" OR "KRAS" OR "RET" OR "ROS1" OR "VEGF" OR "MET" OR "BRAF" OR "ERBB2" OR "HER2" OR "PD-1" OR "PD-L1" OR "target" OR "targeted" OR "TKI" OR “molecular” OR “kinase” OR “monoclonal” OR “antibody” OR “immunotherapy”) AND ("texture" OR "textural" OR "radiomic" OR "radiomics" OR “imaging features” OR “imaging biomarker” OR “imaging biomarkers” OR “imaging characteristics” OR “deep learning” OR “CNN” OR “convolutional”)) AND ("lung" OR "NSCLC" OR "pulmonary")

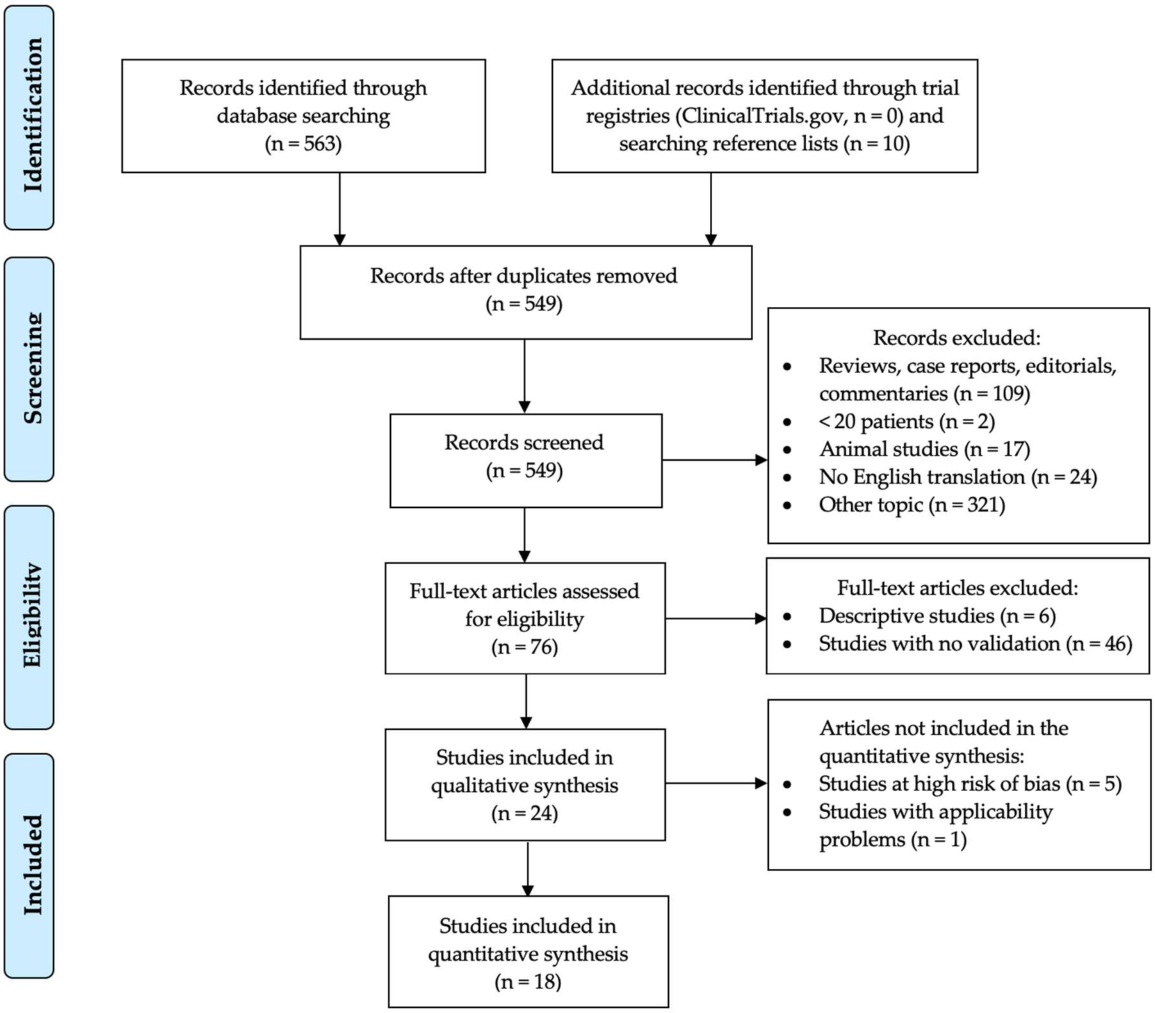

Figure S1. Flow chart of the literature selection process.

Study	RISK OF BIAS: Patient Selection	RISK OF BIAS: Index Test	RISK OF BIAS: Reference Standard	RISK OF BIAS: Flow and Timing	APPLICABILITY CONCERNS: Patient Selection	APPLICABILITY CONCERNS: Index Test	APPLICABILITY CONCERNS: Reference Standard	QUADAS-2 ADHERENCE (0-7) [1]
Yoon 2020	Low	Low	Low	Low	Low	Low	Low	7
Zhao W 2020	Low	Low	Low	Low	Low	Low	Low	7
Lu X 2020	Low	Low	Low	Low	Low	Low	Low	7
Zhang 2019	Low	Unclear	Low	Low	Low	Low	Low	6
Li X 2019	Low	Unclear	Low	Low	Low	Low	Low	6
Koyasu 2020	Unclear	Unclear	Low	Unclear	Low	Low	Low	4
Wang X 2019	Unclear	Unclear	Unclear	Unclear	Low	Low	Low	3
Li S 2019	Low	Low	Unclear	Unclear	High	Low	Low	4
Jiang 2019	Unclear	Low	Low	Low	Low	Low	Low	6
Jiang 2020	Low	Unclear	Low	Low	Low	Low	Low	6
Tu 2019	Low	Low	Unclear	Unclear	Low	Low	Low	5
Zhao W 2019	Unclear	Unclear	Low	Low	Low	Low	Low	5
Yang X 2019	Low	Low	Low	Low	Low	Low	Low	7
Jia 2019	Low	Unclear	Low	Low	Low	Low	Low	6
Li XY 2018	Unclear	Unclear	Low	Low	Low	Low	Low	5
Wang S 2019	Unclear	Low	Low	Low	Low	Low	Low	6
Rizzo 2019	Unclear	Unclear	Unclear	Unclear	Low	Low	Low	3
Li Y 2019	Unclear	Low	Low	Low	Low	Low	Low	6
Xiong JF 2019	Unclear	Low	Low	Low	Low	Low	Low	6
Zhang 2018	Low	Unclear	Low	Low	Low	Low	Low	6
Rios V. 2017	Unclear	Unclear	Low	High	Low	Low	Low	4
Yoon 2015	Unclear	Unclear	Low	Low	Low	Low	Low	5
Gevaert 2017	Unclear	Unclear	Low	Unclear	Low	Low	Low	4
Yamamoto 2014	Unclear	Low	Low	Low	Low	Low	Low	6

Low	11	11	20	17	Low	23	24	24	QUADAS-2 > 4	18
High	0	0	0	1	High	1	0	0	QUADAS-2 ≤ 4	6
Unclear	13	13	4	6	Unclear	0	0	0		

Figure S2. QUADAS-2 tool for quality assessment of each included study. [1] To calculate the adherence to the QUADAS-2 criteria, 1 point was given for every item rated "Low", and 0 points for every item rated "Unclear" or "High". Finally, points for each of the seven items were summed.

Table S1. Details of molecular genetic alterations or PD-L1 expression stratified according to the stage (early vs. advanced) in the "high-quality" papers.

Study	Stage 0–II	Stage III–IV
**EGFR**		
Gevaert et al. [1]	nr	nr
Jia et al. [2]	311 pts (61% EGFR+)	192 pts (60% EGFR+)
Jiang et al. [3]	nr	nr
Koyasu et al. [4]	nr	nr
Li et al. [5]	356 pts (58% EGFR+)	654 pts (47% EGFR+)
Li et al. [6]	14 pts (57% EGFR+)	37 pts (41% EGFR+)
Li et al. [7]	nr	nr
Li et al. [8]	90 pts (56% EGFR+)	25 pts (56% EGFR+)
Lu et al. [9]	83 pts (65% EGFR+)	21 pts (48% EGFR+)
Rios Velazquez et al. [10]	nr	nr
Rizzo et al. [11]	nr	nr
Tu et al. [12]	326 pts (51% EGFR+)	78 pts (27% EGFR+)
Wang et al. [13]	620 pts (55% EGFR+)	224 pts (60% EGFR+)
Wang et al. [14]	51 pts (64% EGFR+)	0
Xiong et al. [15]	311 pts (61% EGFR+)	192 pts (60% EGFR+)
Yang et al. [16]	nr	nr
Zhang et al. [17]	0	180 pts (48% EGFR+)
Zhang et al. [18]	141 pts (59% EGFR+)	107 pts (48% EGFR+)
Zhao et al. [19]	394 pts (nr % EGFR+)	185 pts (nr % EGFR+)
Zhao et al. [20]	430 pts (nr % EGFR+)	207 pts (nr % EGFR+)
**ALK**		
Yamamoto et al. [21]	nr	nr
**ALK/ROS1/RET**		
Yoon et al. [22]	305 pts (9% Fusion+)	232 pts (15% Fusion+)
**PD-L1**		
Jiang et al. [23]	301 pts (20% PD-L1 ≥50%)	98 pts (31% PD-L1 ≥50%)
Yoon et al. [24]	0	153 pts (35% PD-L1 ≥50%)

nr: not reported; na: not assessed; pts: patients.

Table S2. Summary of the study reporting AUC and 95% confidence interval to predict genetic alterations and immunotherapy targets.

Study	Method	Metric(s)-Validation Set	95% Confidence Interval
**EGFR**			
Yang et al. [16]	Radiomics	AUC = 0.79	0.71–0.86
	Radiomics + clinical	AUC = 0.83	0.70–0.86
Jia et al. [2]	Radiomics	AUC = 0.80	0.73–0.87
	Radiomics + clinical	AUC = 0.83	0.76–0.89
Li et al. [6]	Radiomics	AUC = 0.83	0.68-0.92
Li et al. [5]	Radiomics	AUC = 0.74	0.67–0.81
	CNN	AUC = 0.81	0.75-0.87
	Radiomics + CNN	AUC = 0.81	0.75-0.87
Xiong et al. [15]	CNN	AUC = 0.78	0.70-0.85
	Radiomics + CNN	AUC = 0.84	0.78-0.90
**ALK**			
Yamamoto et al. [21]	Visual qualitative	Sensitivity = 83%<br>Specificity = 78%	59%-96%<br>68%-86%
**PD-L1**			
Jiang et al. [23]	Radiomics	AUC = 0.97	0.93-1.0
Jiang et al. [23]	Radiomics*	AUC = 0.97	0.93-1.0
	Radiomics**	AUC = 0.91	0.85-0.97
Yoon et al. [24]	Radiomics + clinical	AUC = 0.67	0.58-0.76

* PD-L1 expression level ≥1%; ** PD-L1 expression level ≥50%.

References

1. Gevaert, O.; Echegaray, S.; Khuong, A.; Hoang, C.D.; Shrager, J.B.; Jensen, K.C.; Berry, G.J.; Guo, H.H.; Lau, C.; Plevritis, S.K.; et al. Predictive radiogenomics modeling of EGFR mutation status in lung cancer. *Sci. Rep.* **2017**, *7*, 1–8.
2. Jia, T.Y.; Xiong, J.F.; Li, X.Y.; Yu, W.; Xu, Z.Y.; Cai, X.W.; Ma, J.C.; Ren, Y.C.; Larsson, R.; Zhang, J.; et al. Identifying EGFR mutations in lung adenocarcinoma by noninvasive imaging using radiomics features and random forest modeling. *Eur. Radiol.* **2019**, *29*, 4742–4750.
3. Jiang, M.; Zhang, Y.; Xu, J.; Ji, M.; Guo, Y.; Guo, Y.; Xiao, J.; Yao, X.; Shi, H.; Zeng, M. Assessing EGFR gene mutation status in non-small cell lung cancer with imaging features from PET/CT. *Nucl. Med. Commun.* **2019**, *40*, 842–849.
4. Koyasu, S.; Nishio, M.; Isoda, H.; Nakamoto, Y.; Togashi, K. Usefulness of gradient tree boosting for predicting histological subtype and EGFR mutation status of non-small cell lung cancer on 18F FDG-PET/CT. *Ann. Nucl. Med.* **2020**, *34*, 49–57.
5. Li, X.Y.; Xiong, J.F.; Jia, T.Y.; Shen, T. Le; Hou, R.P.; Zhao, J.; Fu, X.L. Detection of epithelial growth factor receptor (EGFR) mutations on CT images of patients with lung adenocarcinoma using radiomics and/or multi-level residual convolutionary neural networks. *J. Thorac. Dis.* **2018**, *10*, 6624–6635.
6. Li, Y.; Lu, L.; Xiao, M.; Dercle, L.; Huang, Y.; Zhang, Z.; Schwartz, L.H.; Li, D.; Zhao, B. CT Slice Thickness and Convolution Kernel Affect Performance of a Radiomic Model for Predicting EGFR Status in Non-Small Cell Lung Cancer: A Preliminary Study. *Sci. Rep.* **2018**, *8*, 1–10.
7. Li, S.; Ding, C.; Zhang, H.; Song, J.; Wu, L. Radiomics for the prediction of EGFR mutation subtypes in non-small cell lung cancer. *Med. Phys.* **2019**, *46*, 4545–4552.
8. Li, X.; Yin, G.; Zhang, Y.; Dai, D.; Liu, J.; Chen, P.; Zhu, L.; Ma, W.; Xu, W. Predictive Power of a Radiomic Signature Based on 18F-FDG PET/CT Images for EGFR Mutational Status in NSCLC. *Front. Oncol.* **2019**, *9*, 1–11.
9. Lu, X.; Li, M.; Zhang, H.-M.; Hua, S.; Meng, F.; Yang, H.; Li, X.; Cao, D. A novel radiomic nomogram for predicting epidermal growth factor receptor mutation in peripheral lung adenocarcinoma. *Phys. Med. Biol.* **2020**, *65*, 055012.
10. Rios Velazquez, E.; Parmar, C.; Liu, Y.; Coroller, T.P.; Cruz, G.; Stringfield, O.; Ye, Z.; Makrigiorgos, M.; Fennessy, F.; Mak, R.H.; et al. Somatic Mutations Drive Distinct Imaging Phenotypes in Lung Cancer. *Cancer Res.* **2017**, *77*, 3922–3930.
11. Rizzo, S.; Raimondi, S.; de Jong, E.E.C.; van Elmpt, W.; De Piano, F.; Petrella, F.; Bagnardi, V.; Jochems, A.; Bellomi, M.; Dingemans, A.M.; et al. Genomics of non-small cell lung cancer (NSCLC): Association between

CT-based imaging features and EGFR and K-RAS mutations in 122 patients—An external validation. *Eur. J. Radiol.* **2019**, *110*, 148–155.
12. Tu, W.; Sun, G.; Fan, L.; Wang, Y.; Xia, Y.; Guan, Y.; Li, Q.; Zhang, D.; Liu, S.; Li, Z. Radiomics signature: A potential and incremental predictor for EGFR mutation status in NSCLC patients, comparison with CT morphology. *Lung Cancer* **2019**, *132*, 28–35.
13. Wang, S.; Shi, J.; Ye, Z.; Dong, D.; Yu, D.; Zhou, M.; Liu, Y.; Gevaert, O.; Wang, K.; Zhu, Y.; et al. Predicting EGFR mutation status in lung adenocarcinoma on computed tomography image using deep learning. *Eur. Respir. J.* **2019**, *53*, 1800986.
14. Wang, X.; Kong, C.; Xu, W.; Yang, S.; Shi, D.; Zhang, J.; Du, M.; Wang, S.; Bai, Y.; Zhang, T.; et al. Decoding tumor mutation burden and driver mutations in early stage lung adenocarcinoma using CT-based radiomics signature. *Thorac. Cancer* **2019**, *10*, 1904–1912.
15. Xiong, J.-F.; Jia, T.-Y.; Li, X.-Y.; Yu, W.; Xu, Z.-Y.; Cai, X.-W.; Fu, L.; Zhang, J.; Qin, B.-J.; Fu, X.-L.; et al. Identifying epidermal growth factor receptor mutation status in patients with lung adenocarcinoma by three-dimensional convolutional neural networks. *Br. J. Radiol.* **2018**, *91*, 20180334.
16. Yang, X.; Dong, X.; Wang, J.; Li, W.; Gu, Z.; Gao, D.; Zhong, N.; Guan, Y. Computed Tomography-Based Radiomics Signature: A Potential Indicator of Epidermal Growth Factor Receptor Mutation in Pulmonary Adenocarcinoma Appearing as a Subsolid Nodule. *Oncologist* **2019**, *24*, 1156–1164.
17. Zhang, L.; Chen, B.; Liu, X.; Song, J.; Fang, M.; Hu, C.; Dong, D.; Li, W.; Tian, J. Quantitative Biomarkers for Prediction of Epidermal Growth Factor Receptor Mutation in Non-Small Cell Lung Cancer. *Transl. Oncol.* **2018**, *11*, 94–101.
18. Zhang, J.; Zhao, X.; Zhao, Y.; Zhang, J.; Zhang, Z.; Wang, J.; Wang, Y.; Dai, M.; Han, J. Value of pre-therapy 18F-FDG PET/CT radiomics in predicting EGFR mutation status in patients with non-small cell lung cancer. *Eur. J. Nucl. Med. Mol. Imaging* **2020**, *47*, 1137–1146.
19. Zhao, W.; Yang, J.; Ni, B.; Bi, D.; Sun, Y.; Xu, M.; Zhu, X.; Li, C.; Jin, L.; Gao, P.; et al. Toward automatic prediction of EGFR mutation status in pulmonary adenocarcinoma with 3D deep learning. *Cancer Med.* **2019**, *8*, 3532–3543.
20. Zhao, W.; Wu, Y.; Xu, Y.; Sun, Y.; Gao, P.; Tan, M.; Ma, W.; Li, C.; Jin, L.; Hua, Y.; et al. The Potential of Radiomics Nomogram in Non-invasively Prediction of Epidermal Growth Factor Receptor Mutation Status and Subtypes in Lung Adenocarcinoma. *Front. Oncol.* **2020**, *9*.
21. Yamamoto, S.; Korn, R.L.; Oklu, R.; Migdal, C.; Gotway, M.B.; Weiss, G.J.; Iafrate, A.J.; Kim, D.W.; Kuo, M.D. ALK molecular phenotype in non-small cell lung cancer: CT radiogenomic characterization. *Radiology* **2014**, *272*, 568–576.
22. Yoon, H.J.; Sohn, I.; Cho, J.H.; Lee, H.Y.; Kim, J.H.; Choi, Y. La; Kim, H.; Lee, G.; Lee, K.S.; Kim, J. Decoding tumor phenotypes for ALK, ROS1, and RET fusions in lung adenocarcinoma using a radiomics approach. *Med. (United States)* **2015**, *94*, 1–8.
23. Jiang, M.; Sun, D.; Guo, Y.; Guo, Y.; Xiao, J.; Wang, L.; Yao, X. Assessing PD-L1 Expression Level by Radiomic Features From PET/CT in Nonsmall Cell Lung Cancer Patients: An Initial Result. *Acad. Radiol.* **2020**, *27*, 171–179.
24. Yoon, J.; Suh, Y.J.; Han, K.; Cho, H.; Lee, H.; Hur, J.; Choi, B.W. Utility of CT radiomics for prediction of PD-L1 expression in advanced lung adenocarcinomas. *Thorac. Cancer* **2020**, *11*, 993–1004.
